# Supplementary material for: Hepatic PTEN Signaling Regulates Systemic Metabolic Homeostasis through Hepatokines-Mediated Liver-to-Peripheral Organs Crosstalk
Source: Int J Mol Sci. 2022 Apr 2;23(7):3959. doi: 10.3390/ijms23073959 (PMC8999584; doi:10.3390/ijms23073959)
Supplement: Supplementary file 1 [file ijms-23-03959-s001.zip › Berthou et al. - Supplementary Figures.pptx]

## Slide 1
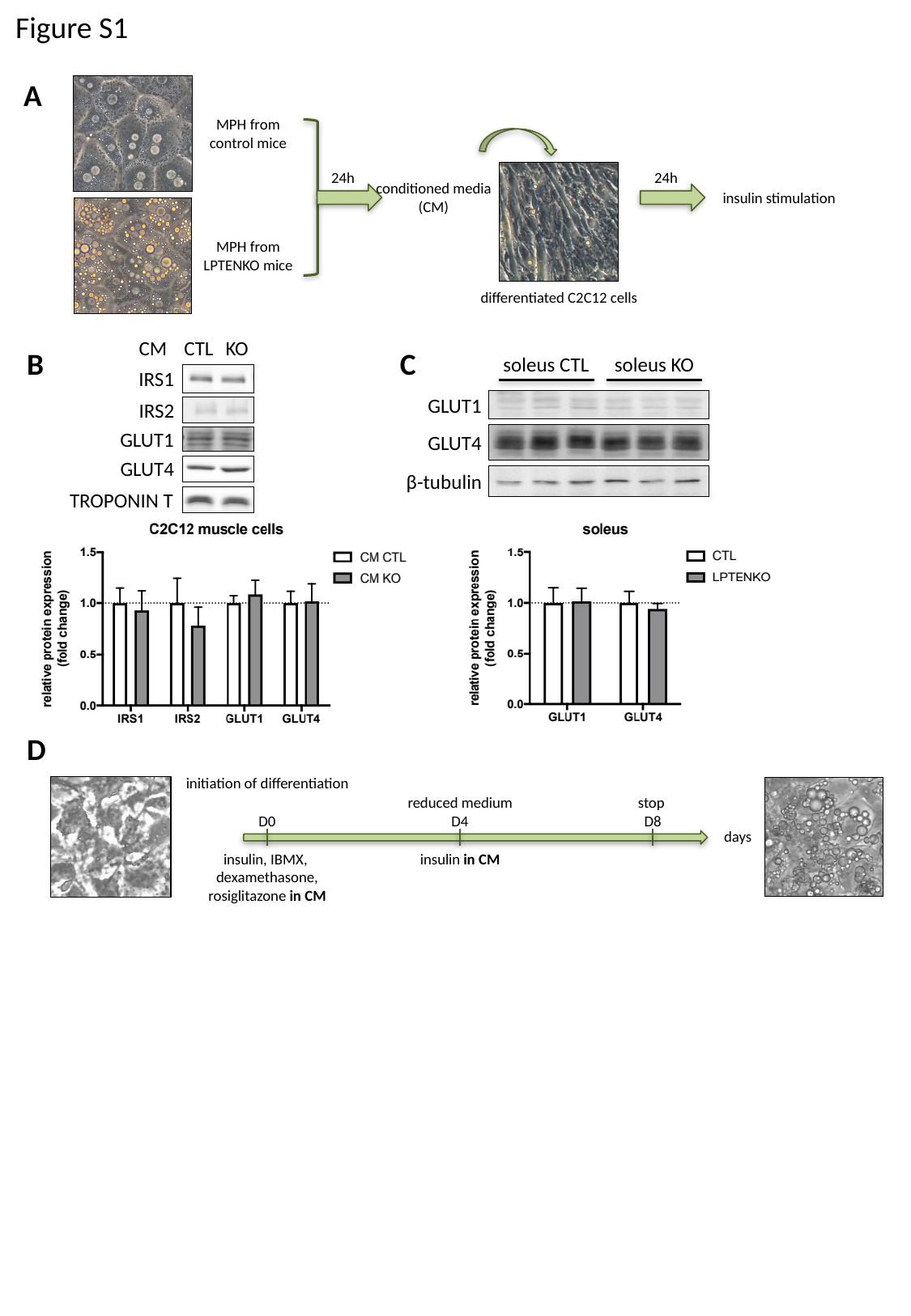

Figure S1
A
MPH from control mice
MPH from LPTENKO mice
24h
differentiated C2C12 cells
24h
conditioned media
(CM)
insulin stimulation
CM
CTL
KO
IRS1
IRS2
GLUT1
GLUT4
TROPONIN T
B
C
soleus CTL
soleus KO
GLUT1
GLUT4
β-tubulin
D
initiation of differentiation
reduced medium
stop
D0
D4
D8
days
insulin, IBMX,
dexamethasone, rosiglitazone in CM
insulin in CM

## Slide 2
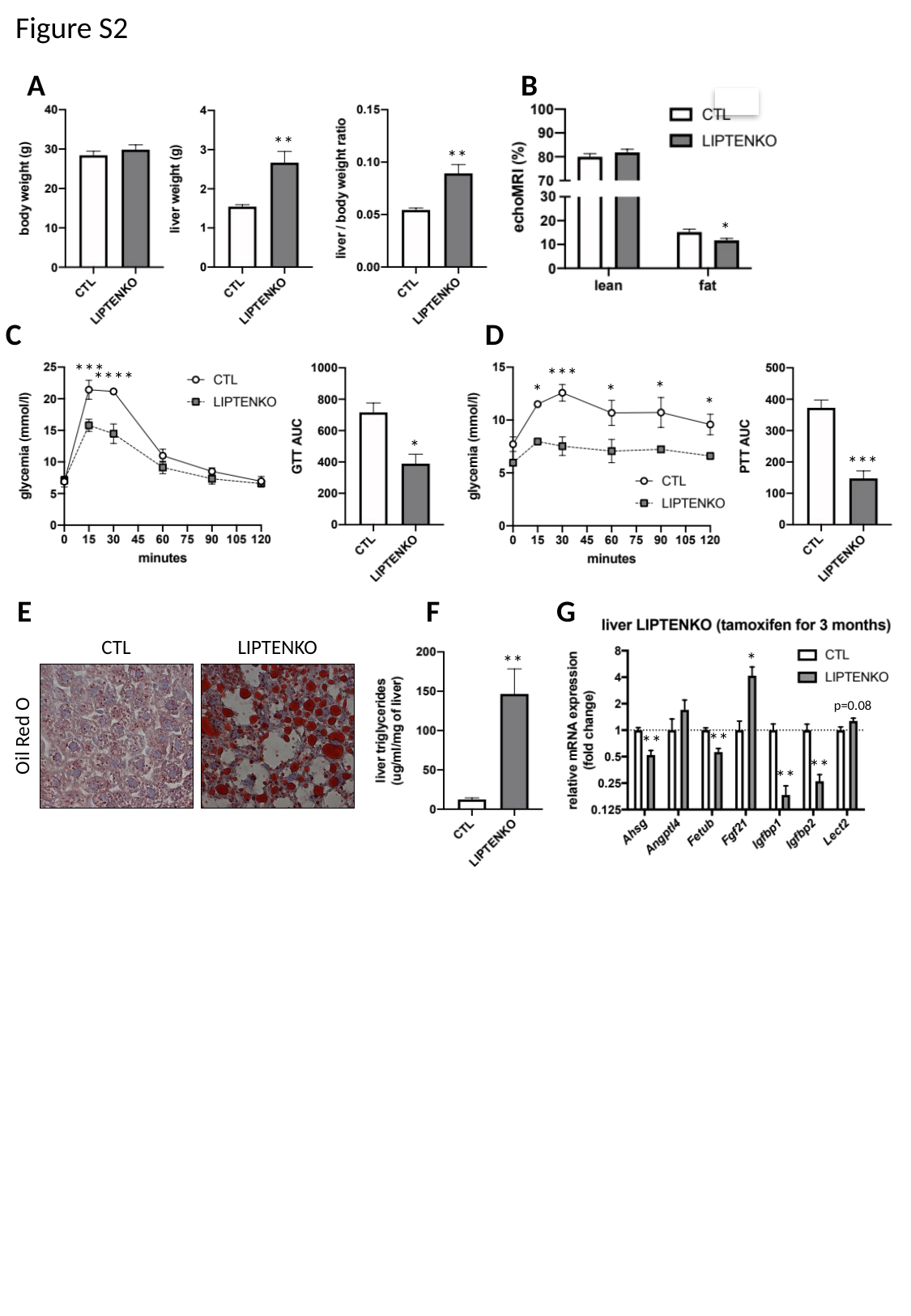

Figure S2
A
B
*
**
**
C
D
***
****
***
*
*
*
*
***
*
E
F
G
*
p=0.08
**
**
**
**
CTL
LIPTENKO
**
Oil Red O

## Slide 3
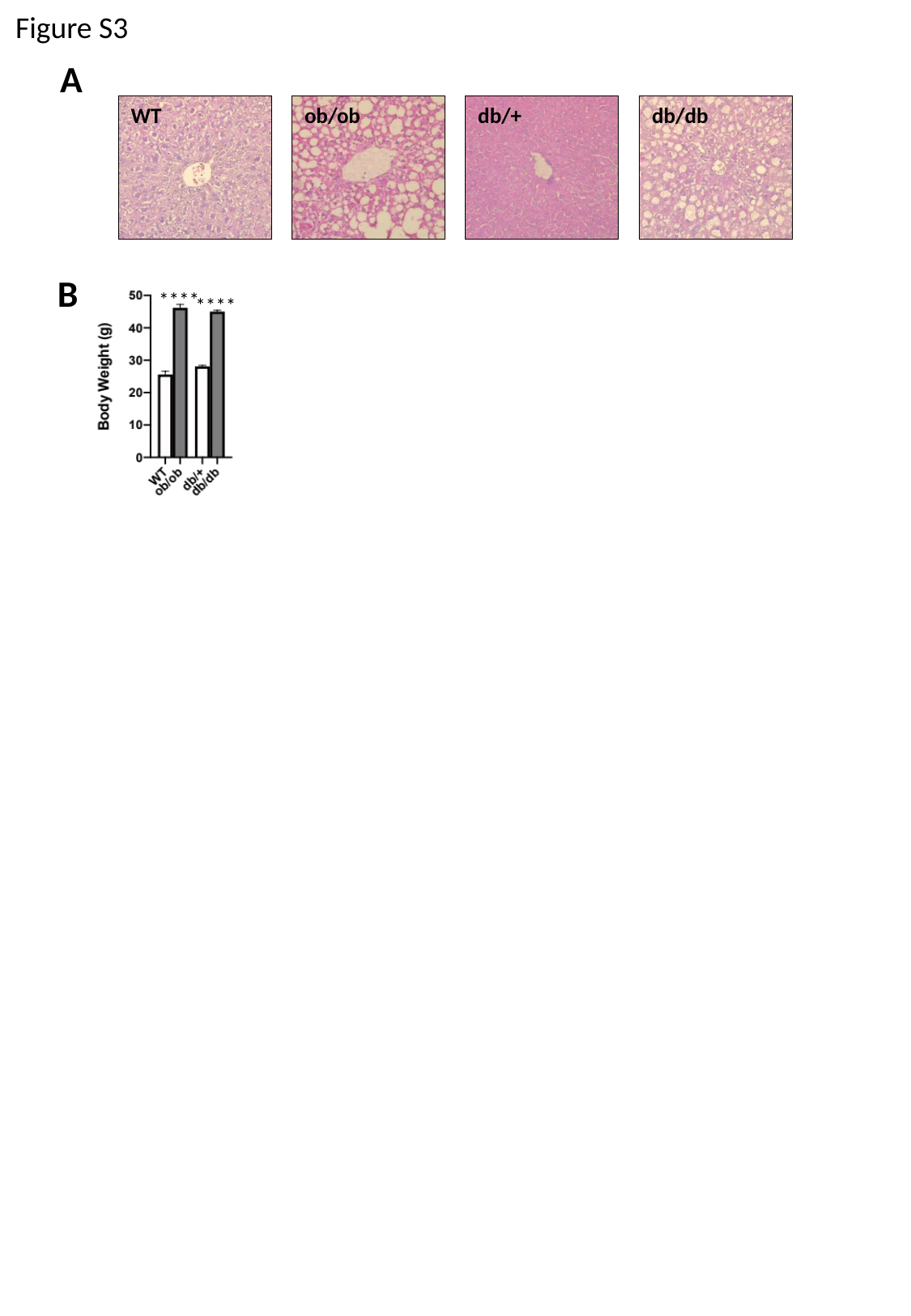

Figure S3
A
WT
ob/ob
db/+
db/db
B
****
****

## Slide 4
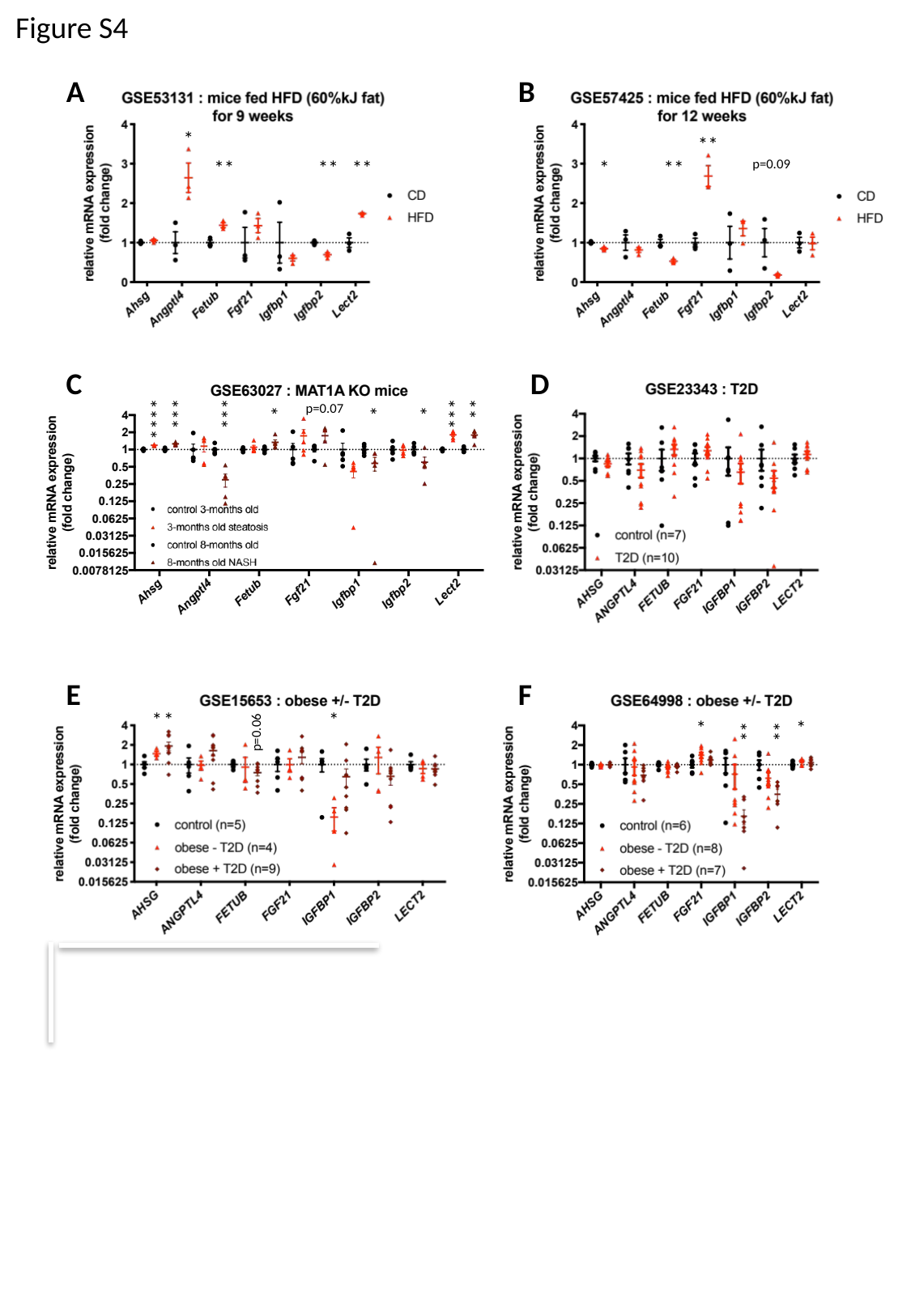

Figure S4
A
B
*
**
**
**
**
*
**
p=0.09
C
D
**
*
*
*
p=0.07
***
***
***
****
E
F
*
*
*
p=0.06
*
*
**
**

## Slide 5
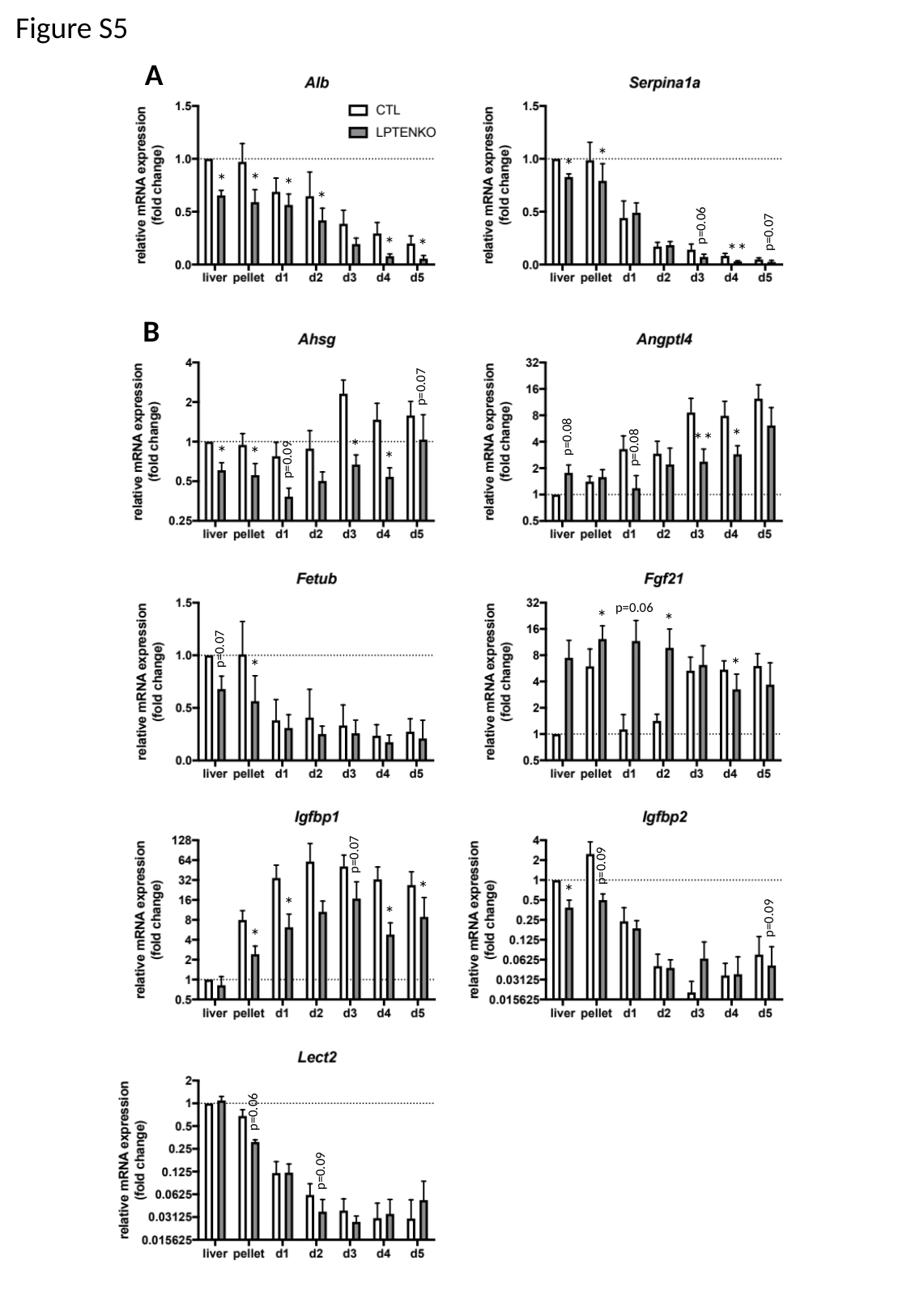

Figure S5
A
*
*
*
*
*
*
*
*
p=0.06
p=0.07
**
B
p=0.07
*
*
*
*
p=0.09
*
**
p=0.08
p=0.08
p=0.07
*
p=0.06
*
*
*
p=0.07
*
*
*
*
p=0.09
*
p=0.09
p=0.06
p=0.09

## Slide 6
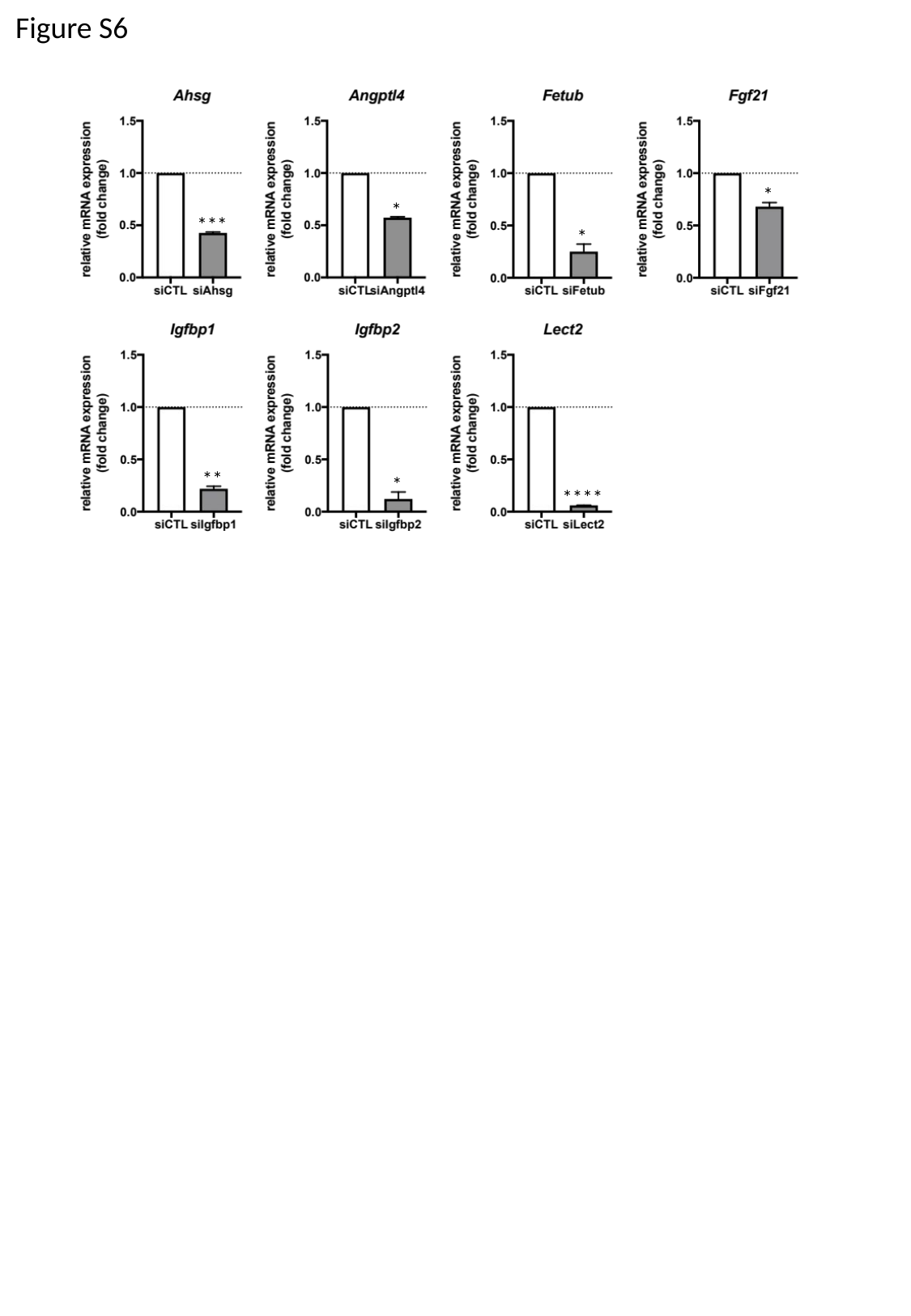

Figure S6
***
*
*
*
**
*
****

## Slide 7
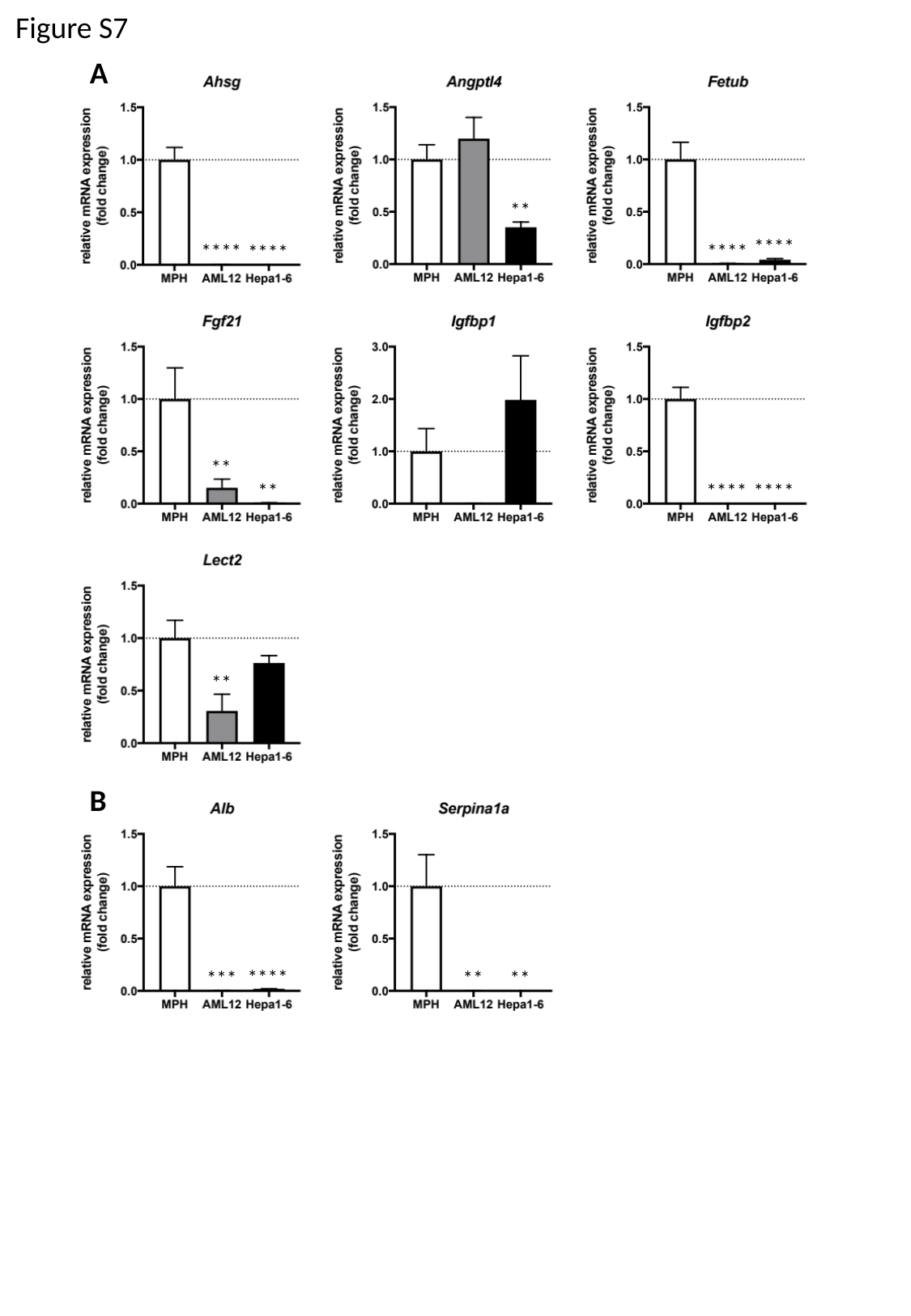

Figure S7
A
**
****
****
****
****
**
**
****
****
**
B
****
**
***
**

## Slide 8
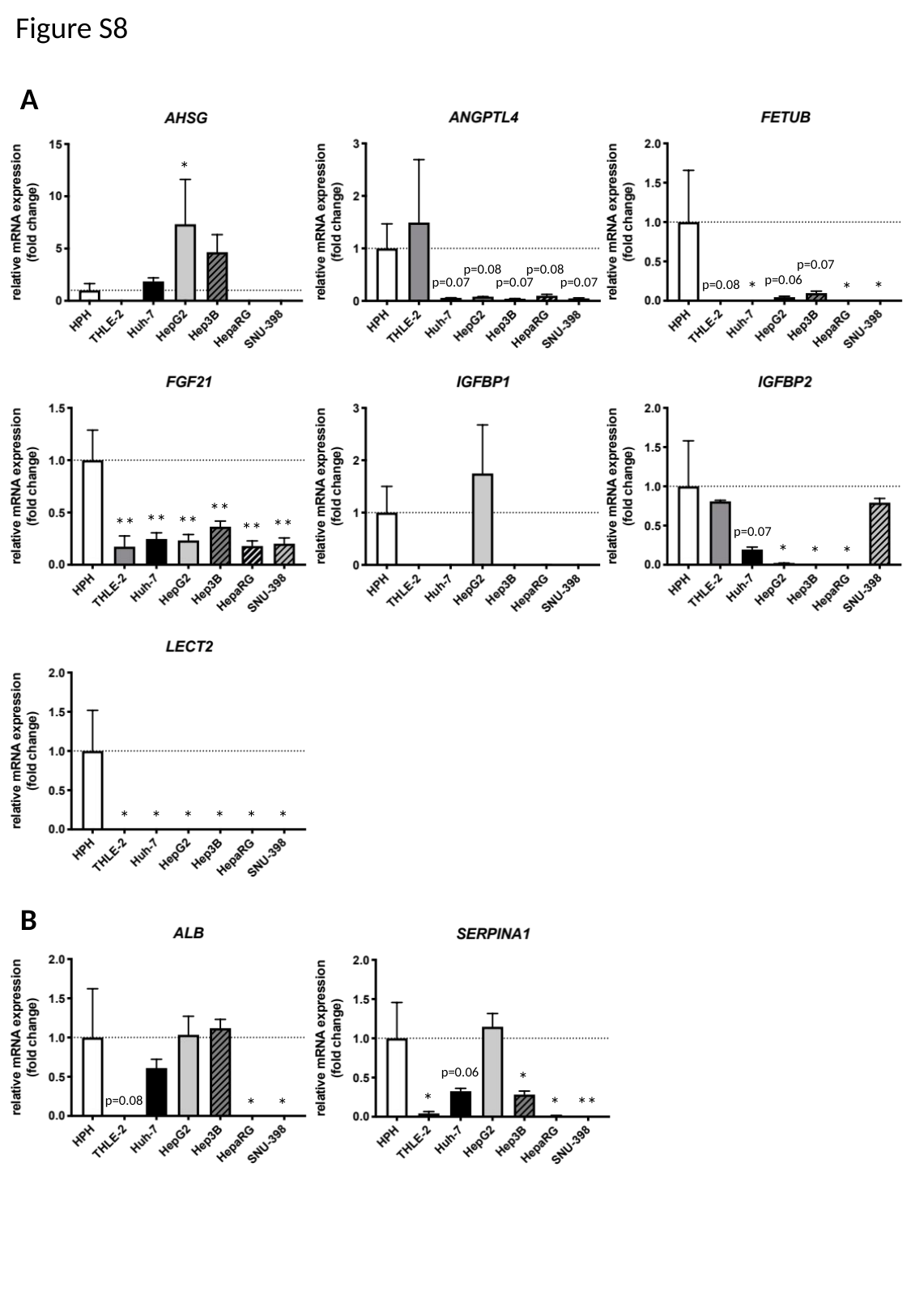

Figure S8
A
p=0.08
p=0.08
p=0.07
p=0.07
p=0.07
p=0.07
p=0.06
p=0.08
*
*
*
*
p=0.07
*
*
*
**
**
**
**
**
**
*
*
*
*
*
*
B
p=0.08
*
*
p=0.06
*
*
*
**

## Slide 9
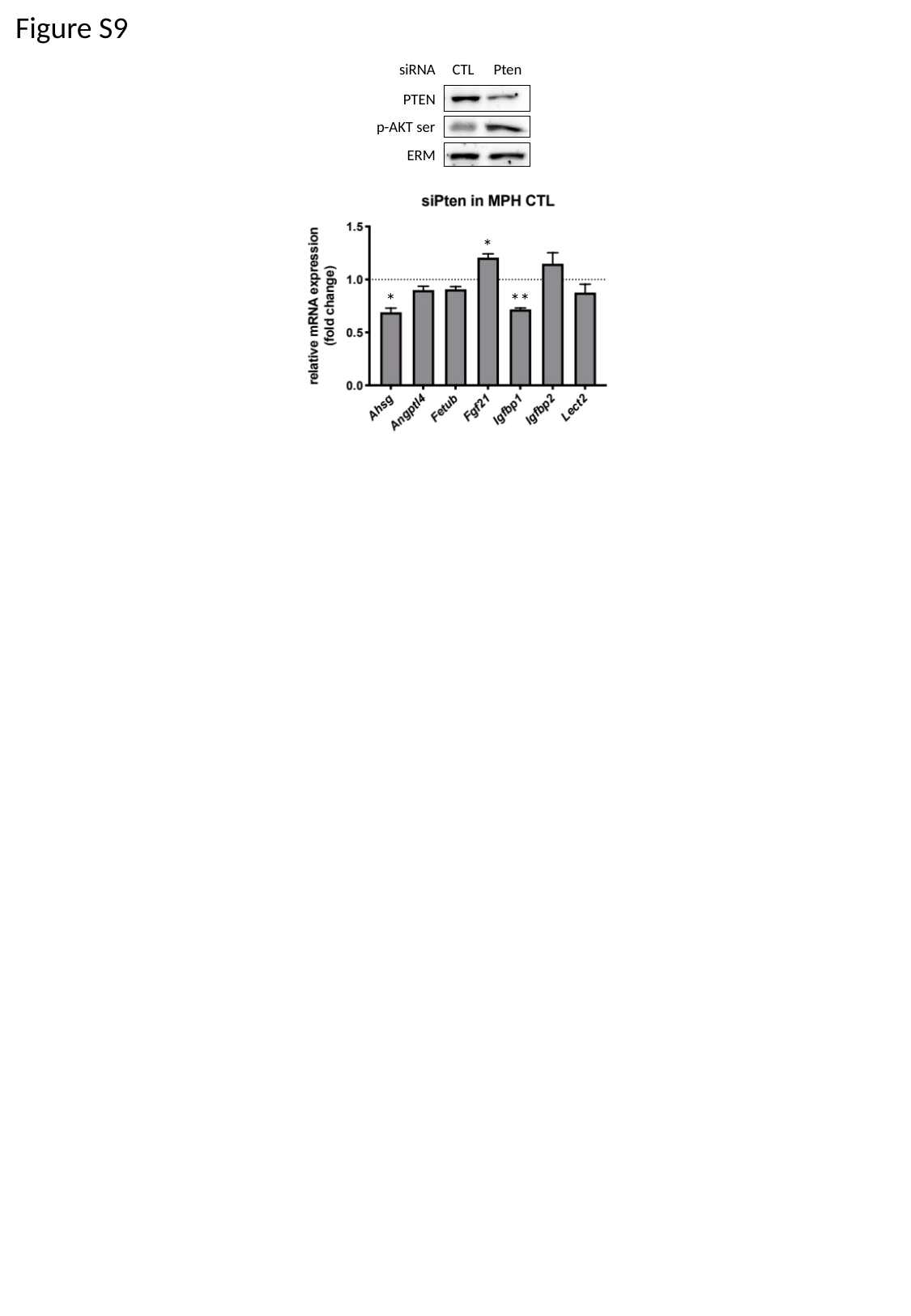

Figure S9
siRNA
CTL
Pten
PTEN
p-AKT ser
ERM
*
**
*

## Slide 10
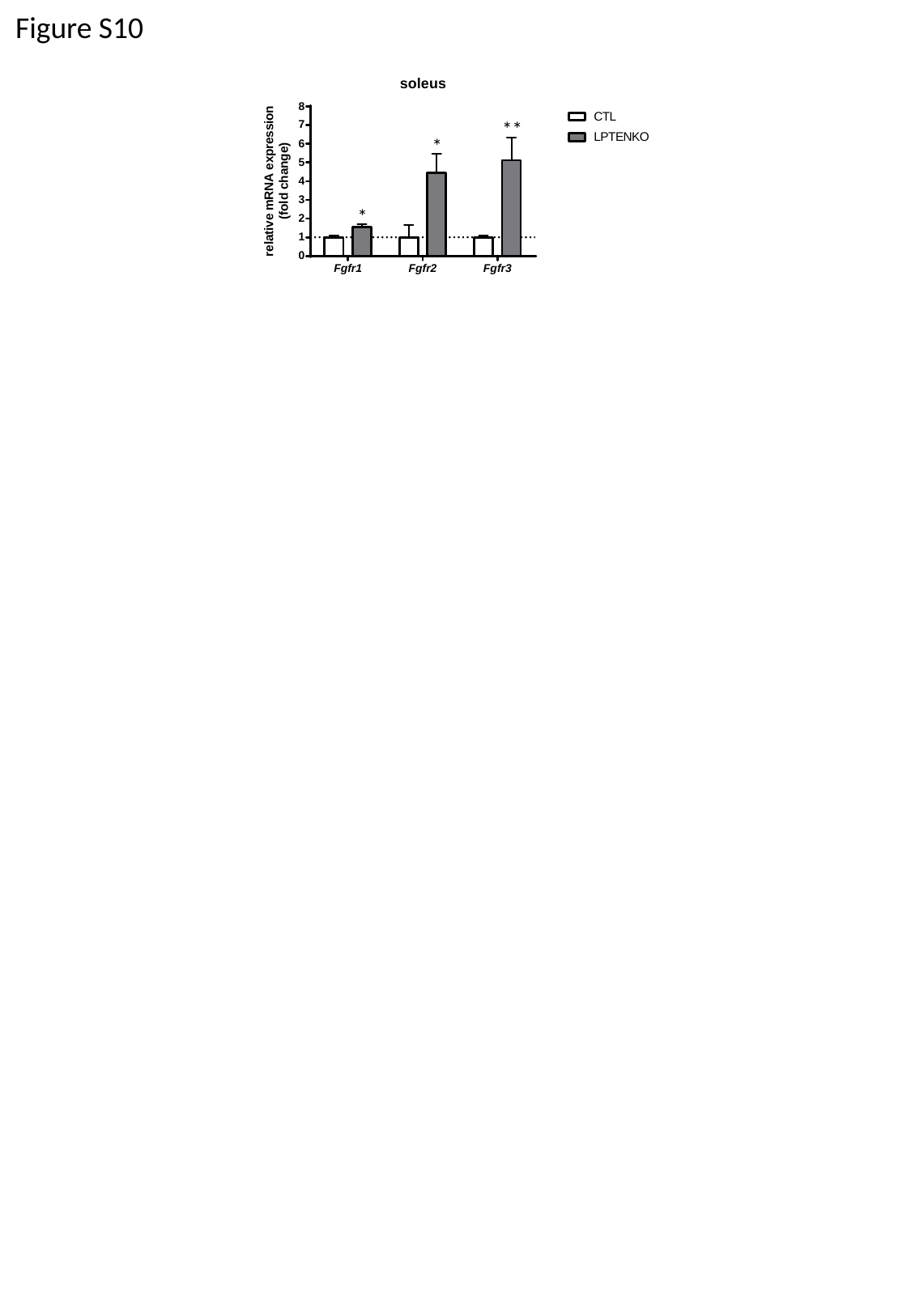

Figure S10
**
*
*

## Slide 11
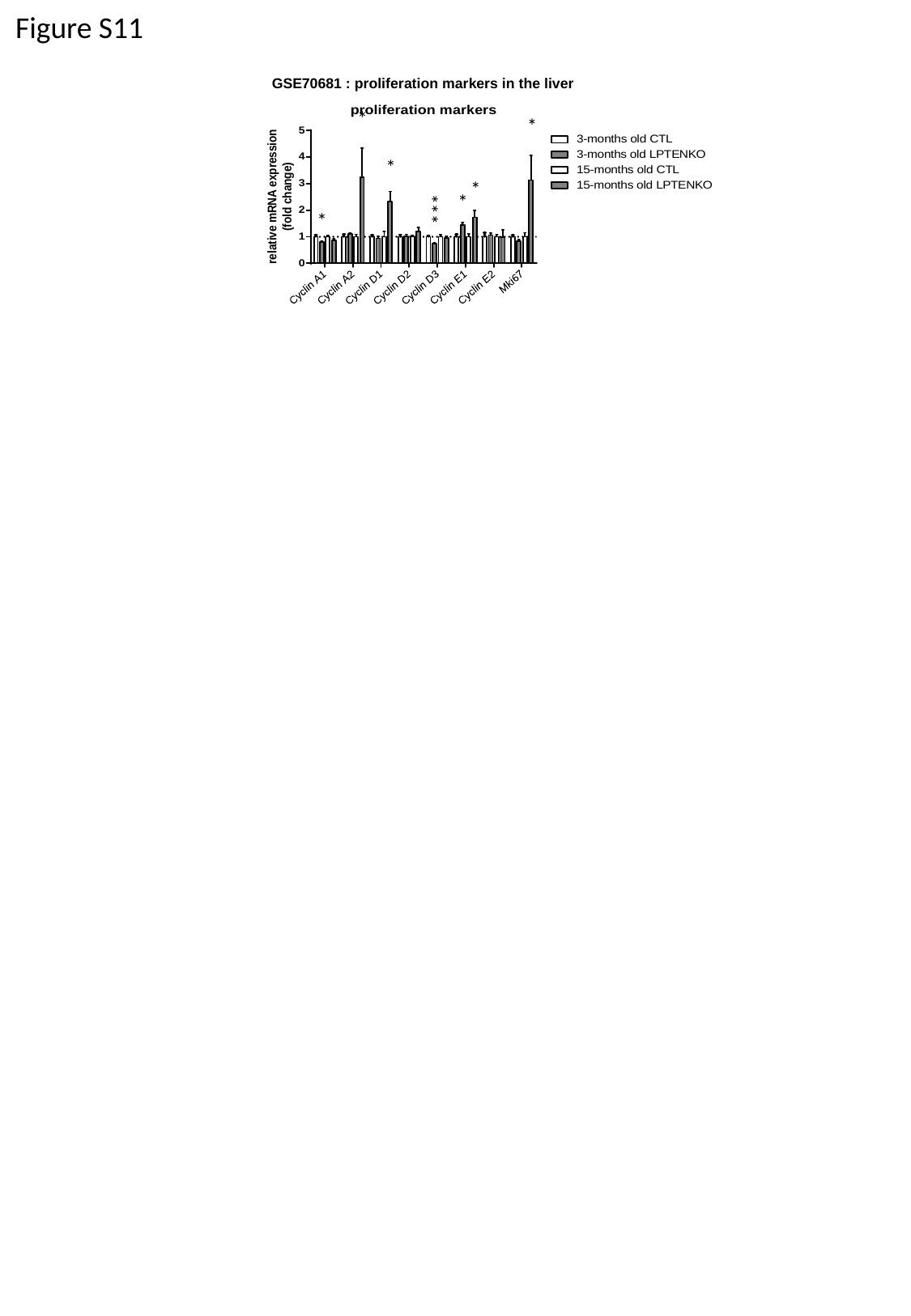

Figure S11
GSE70681 : proliferation markers in the liver
*
*
*
*
*
***
*

## Slide 12
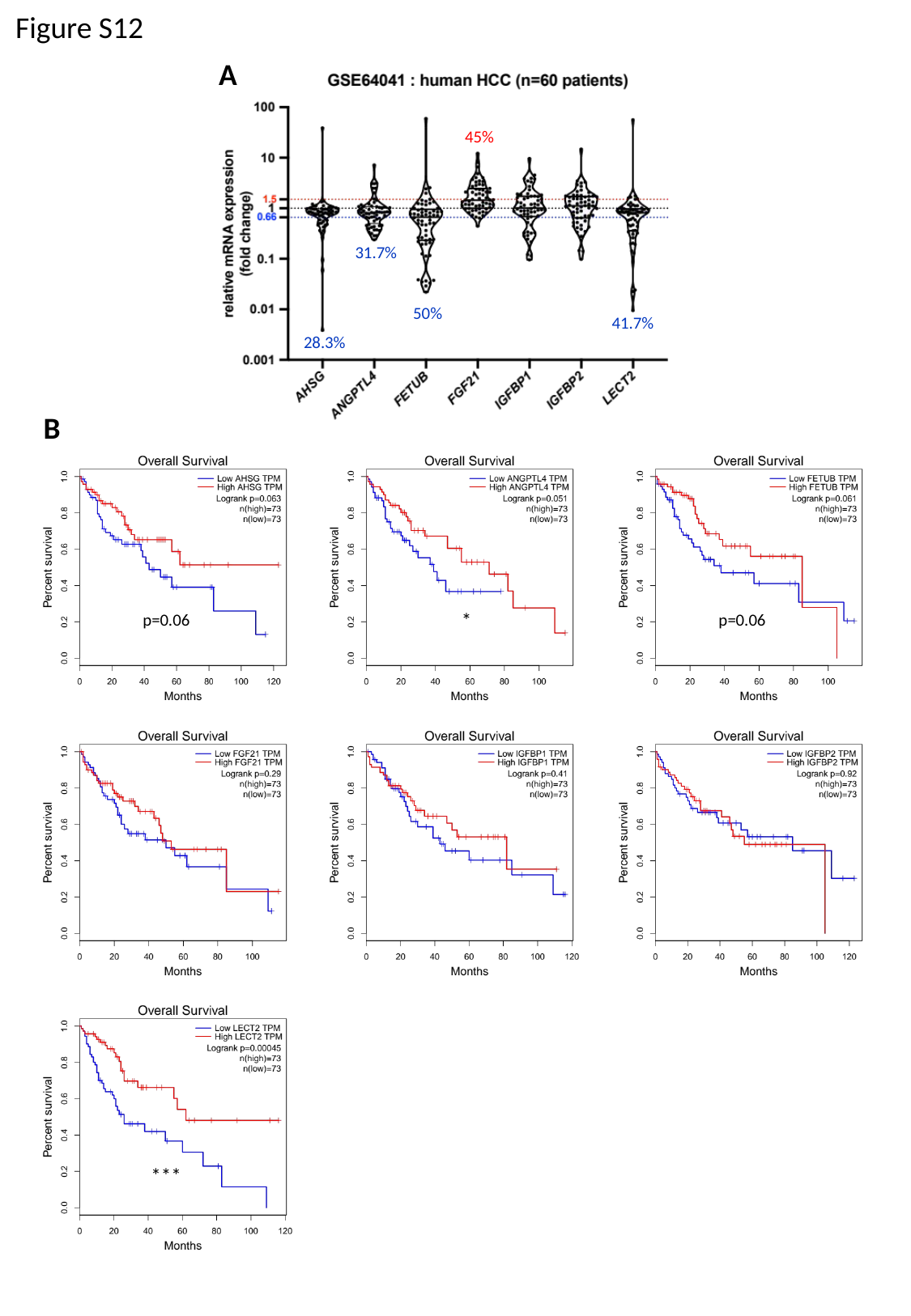

Figure S12
A
45%
31.7%
50%
41.7%
28.3%
B
p=0.06
*
p=0.06
***
