# Supplementary material for: Hepatic PTEN Signaling Regulates Systemic Metabolic Homeostasis through Hepatokines-Mediated Liver-to-Peripheral Organs Crosstalk
Source: Int J Mol Sci. 2022 Apr 2;23(7):3959. doi: 10.3390/ijms23073959 (PMC8999584; doi:10.3390/ijms23073959)
Supplement: Supplementary file 1 [file ijms-23-03959-s001.zip › Berthou et al. - Supplementary Tables.pptx]

## Slide 1
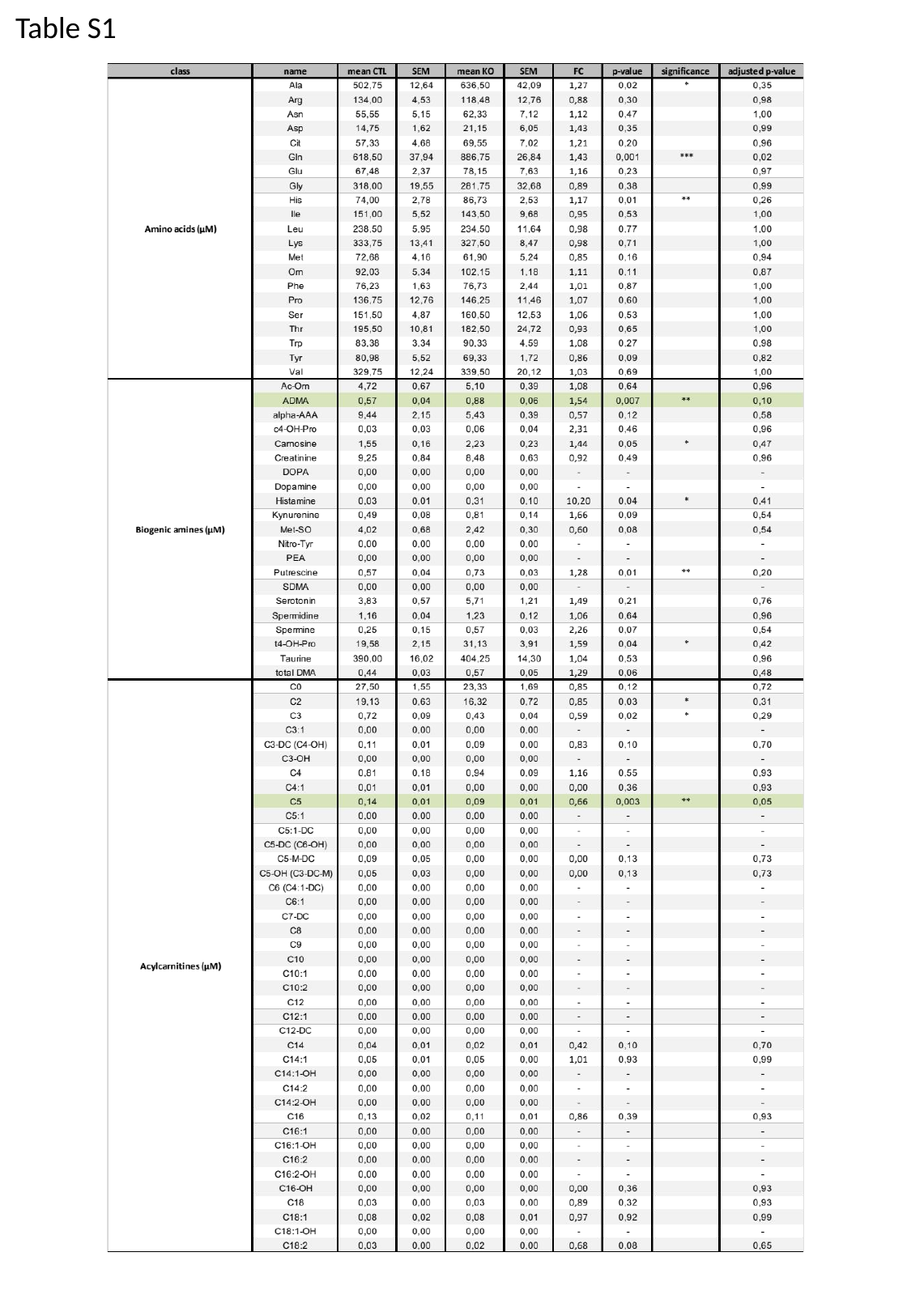

Table S1

## Slide 2
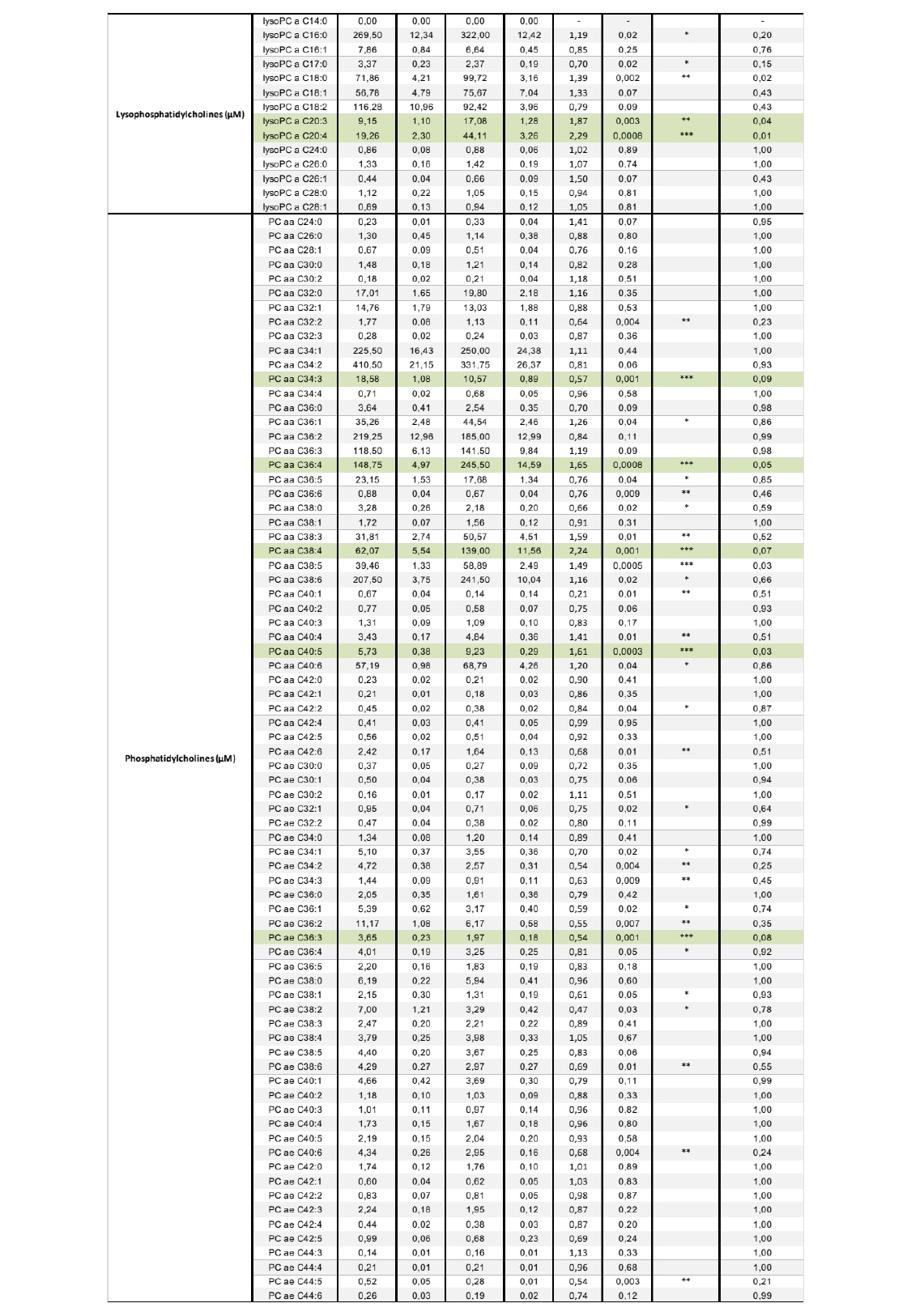

## Slide 3
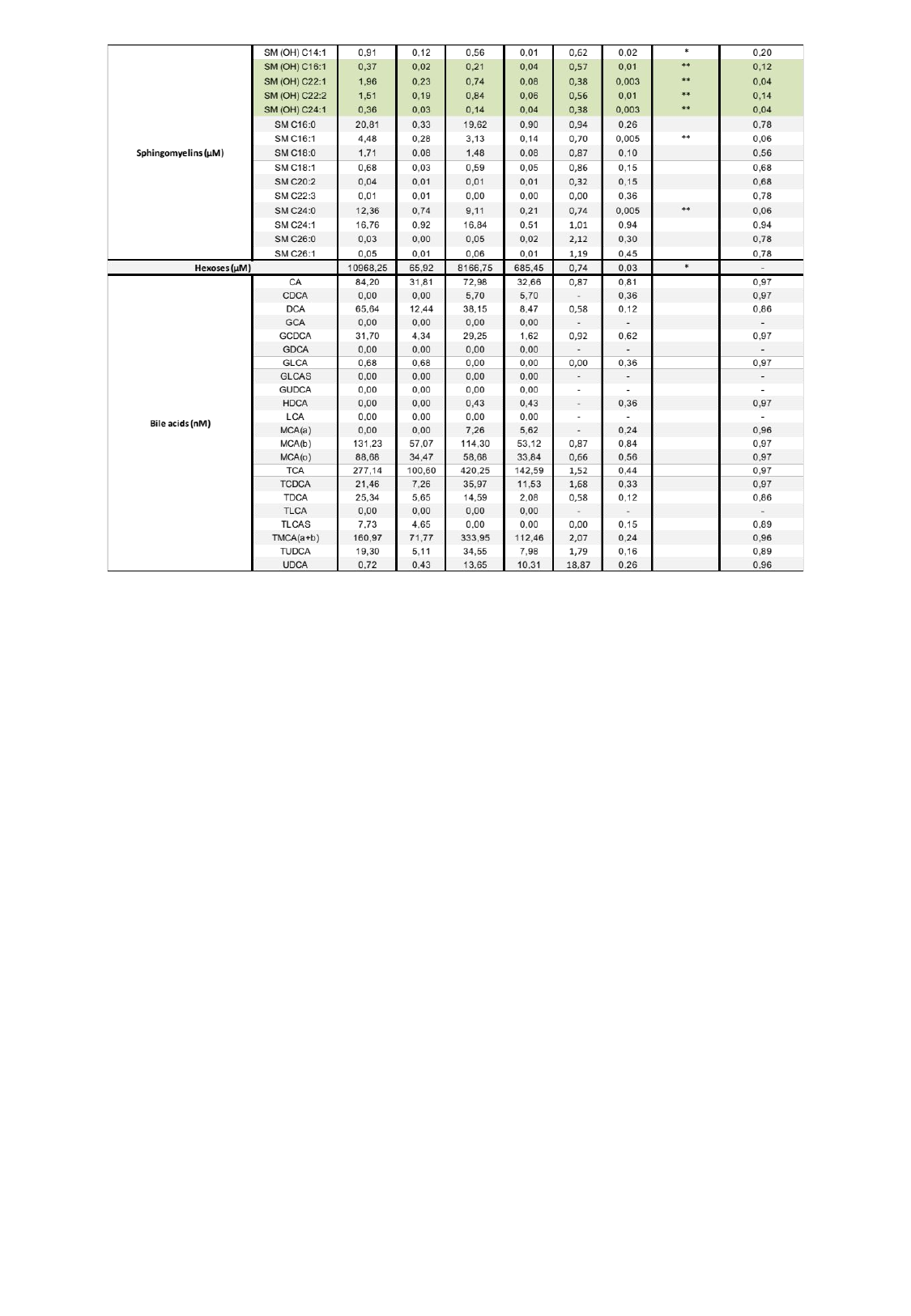

## Slide 4
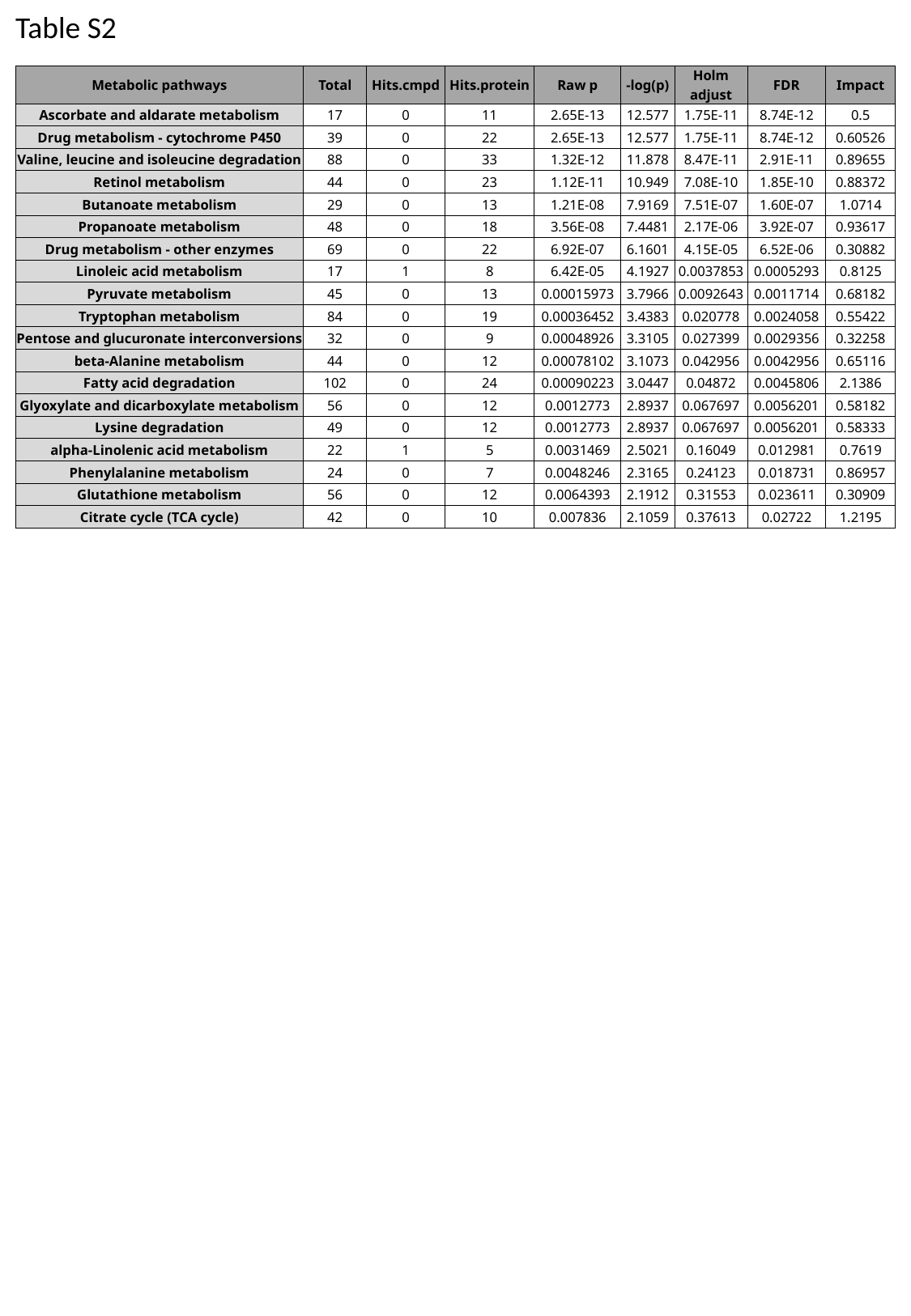

Table S2
| Metabolic pathways | Total | Hits.cmpd | Hits.protein | Raw p | -log(p) | Holm adjust | FDR | Impact |
| --- | --- | --- | --- | --- | --- | --- | --- | --- |
| Ascorbate and aldarate metabolism | 17 | 0 | 11 | 2.65E-13 | 12.577 | 1.75E-11 | 8.74E-12 | 0.5 |
| Drug metabolism - cytochrome P450 | 39 | 0 | 22 | 2.65E-13 | 12.577 | 1.75E-11 | 8.74E-12 | 0.60526 |
| Valine, leucine and isoleucine degradation | 88 | 0 | 33 | 1.32E-12 | 11.878 | 8.47E-11 | 2.91E-11 | 0.89655 |
| Retinol metabolism | 44 | 0 | 23 | 1.12E-11 | 10.949 | 7.08E-10 | 1.85E-10 | 0.88372 |
| Butanoate metabolism | 29 | 0 | 13 | 1.21E-08 | 7.9169 | 7.51E-07 | 1.60E-07 | 1.0714 |
| Propanoate metabolism | 48 | 0 | 18 | 3.56E-08 | 7.4481 | 2.17E-06 | 3.92E-07 | 0.93617 |
| Drug metabolism - other enzymes | 69 | 0 | 22 | 6.92E-07 | 6.1601 | 4.15E-05 | 6.52E-06 | 0.30882 |
| Linoleic acid metabolism | 17 | 1 | 8 | 6.42E-05 | 4.1927 | 0.0037853 | 0.0005293 | 0.8125 |
| Pyruvate metabolism | 45 | 0 | 13 | 0.00015973 | 3.7966 | 0.0092643 | 0.0011714 | 0.68182 |
| Tryptophan metabolism | 84 | 0 | 19 | 0.00036452 | 3.4383 | 0.020778 | 0.0024058 | 0.55422 |
| Pentose and glucuronate interconversions | 32 | 0 | 9 | 0.00048926 | 3.3105 | 0.027399 | 0.0029356 | 0.32258 |
| beta-Alanine metabolism | 44 | 0 | 12 | 0.00078102 | 3.1073 | 0.042956 | 0.0042956 | 0.65116 |
| Fatty acid degradation | 102 | 0 | 24 | 0.00090223 | 3.0447 | 0.04872 | 0.0045806 | 2.1386 |
| Glyoxylate and dicarboxylate metabolism | 56 | 0 | 12 | 0.0012773 | 2.8937 | 0.067697 | 0.0056201 | 0.58182 |
| Lysine degradation | 49 | 0 | 12 | 0.0012773 | 2.8937 | 0.067697 | 0.0056201 | 0.58333 |
| alpha-Linolenic acid metabolism | 22 | 1 | 5 | 0.0031469 | 2.5021 | 0.16049 | 0.012981 | 0.7619 |
| Phenylalanine metabolism | 24 | 0 | 7 | 0.0048246 | 2.3165 | 0.24123 | 0.018731 | 0.86957 |
| Glutathione metabolism | 56 | 0 | 12 | 0.0064393 | 2.1912 | 0.31553 | 0.023611 | 0.30909 |
| Citrate cycle (TCA cycle) | 42 | 0 | 10 | 0.007836 | 2.1059 | 0.37613 | 0.02722 | 1.2195 |

## Slide 5
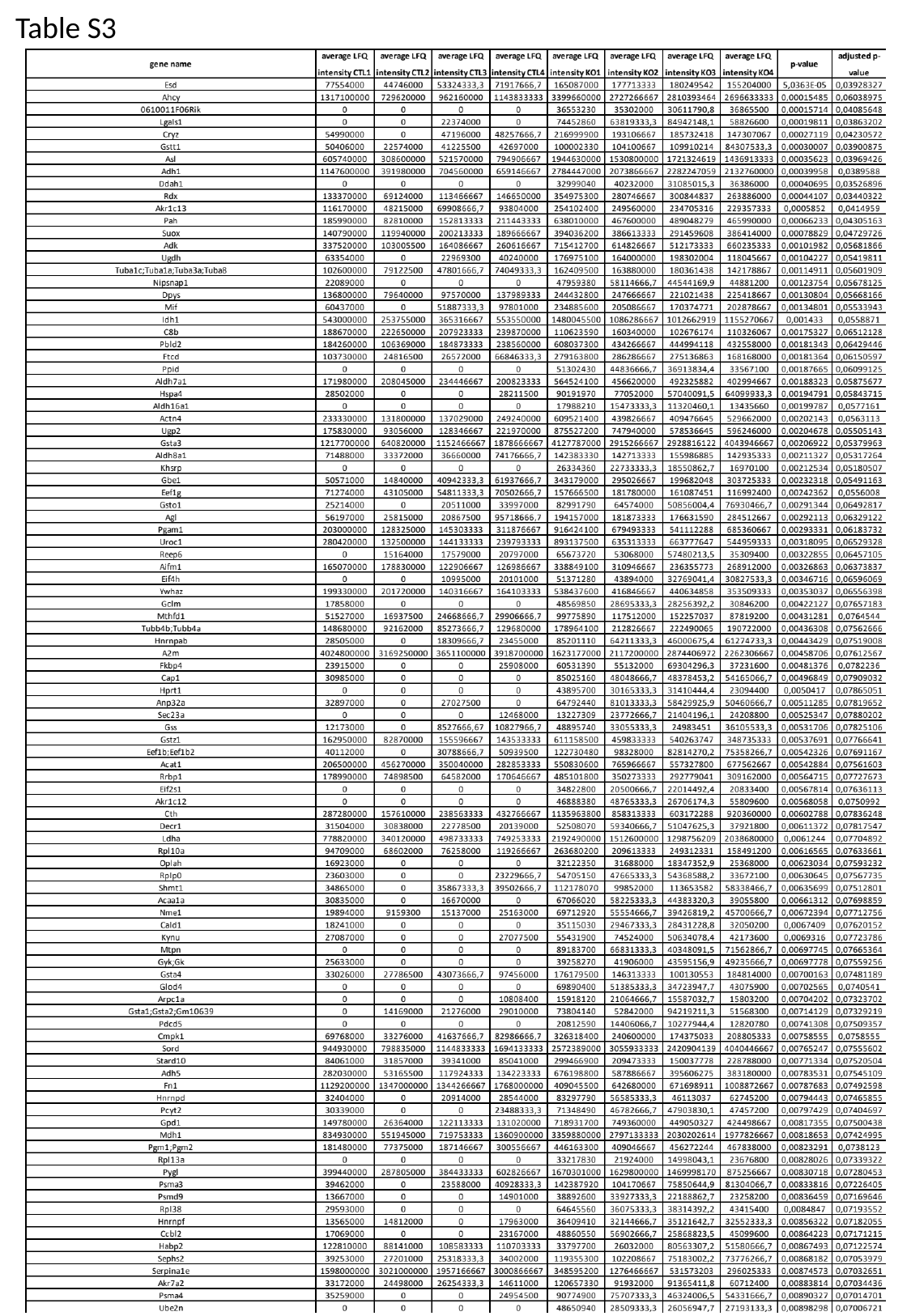

Table S3

## Slide 6
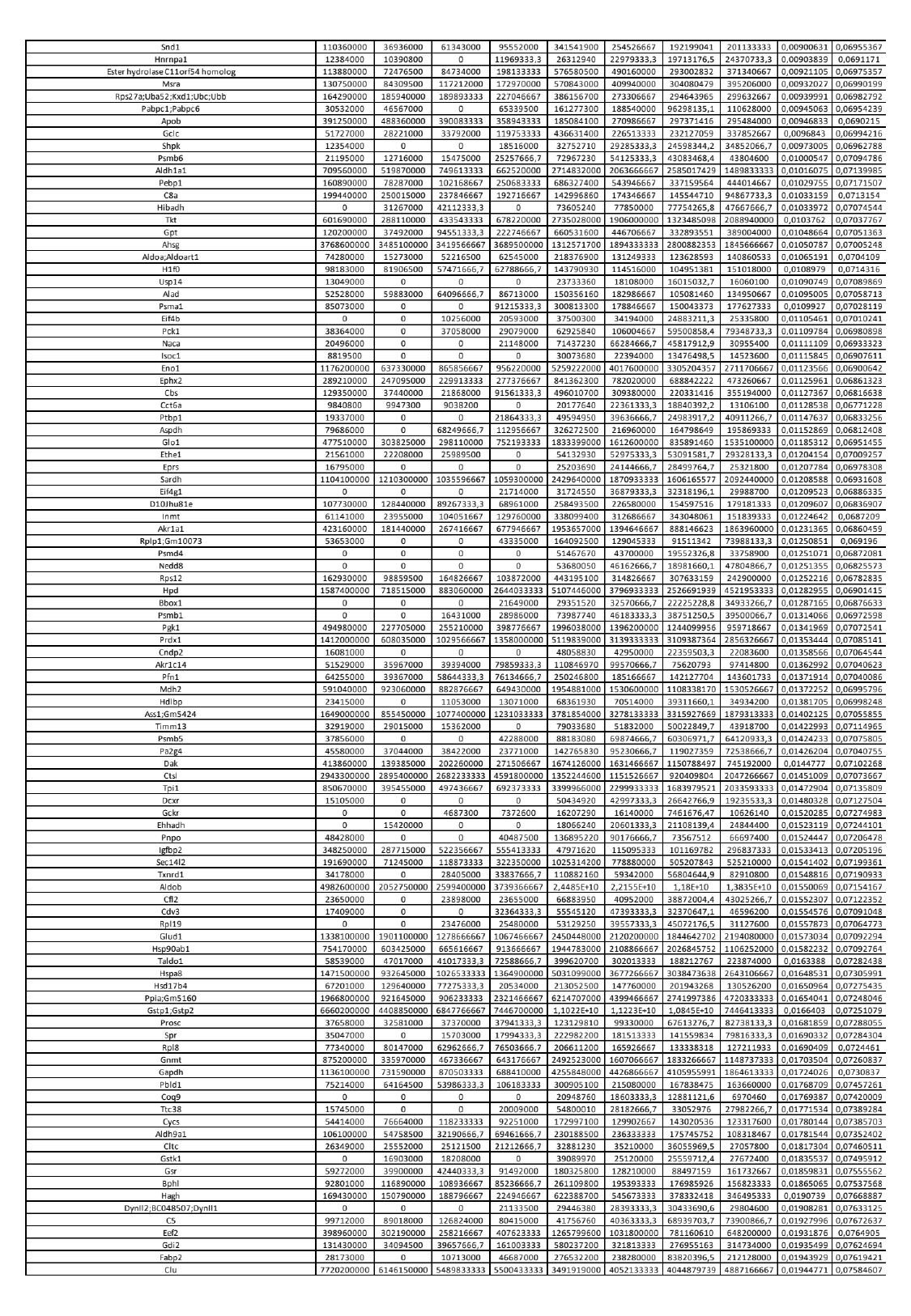

## Slide 7
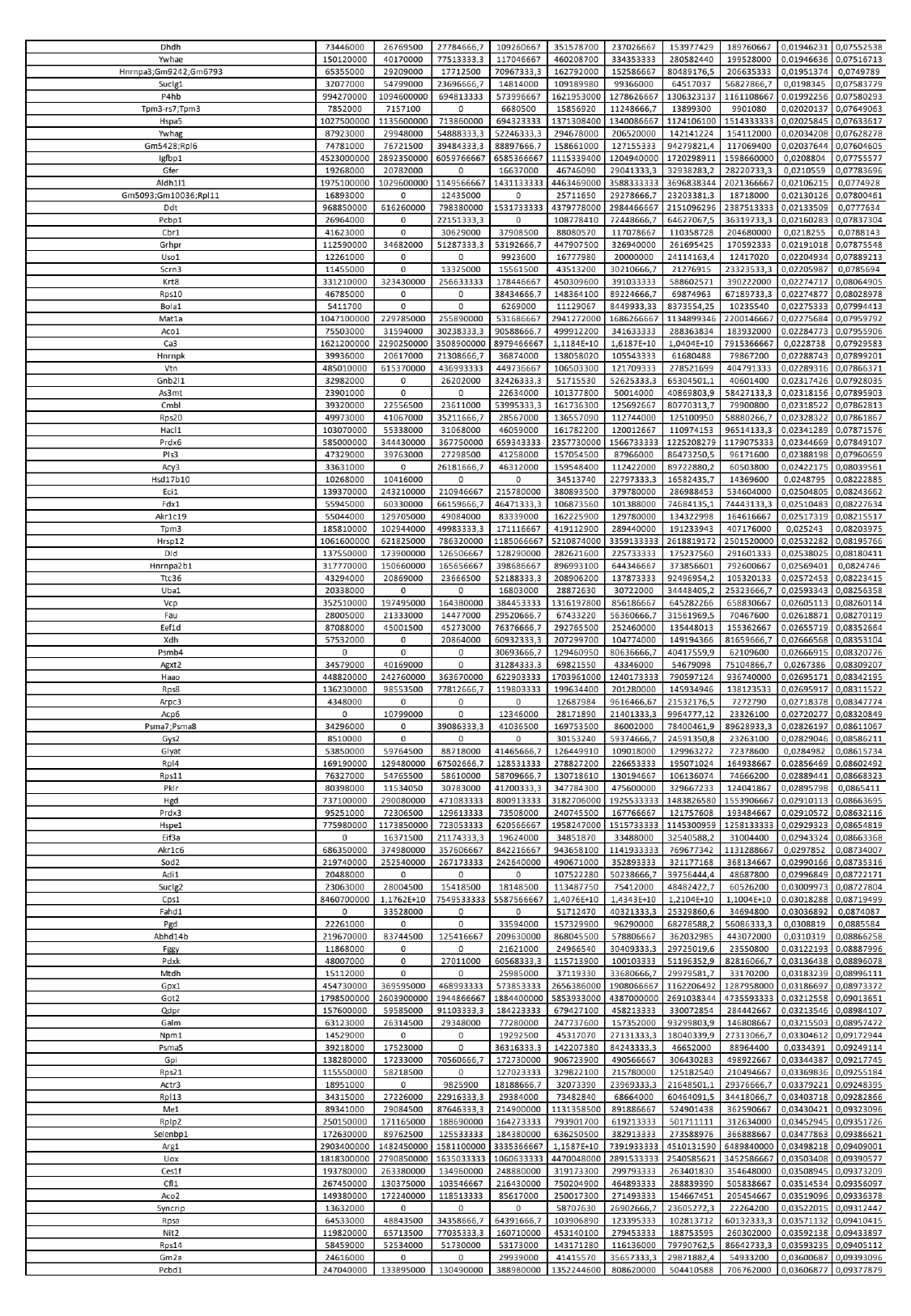

## Slide 8
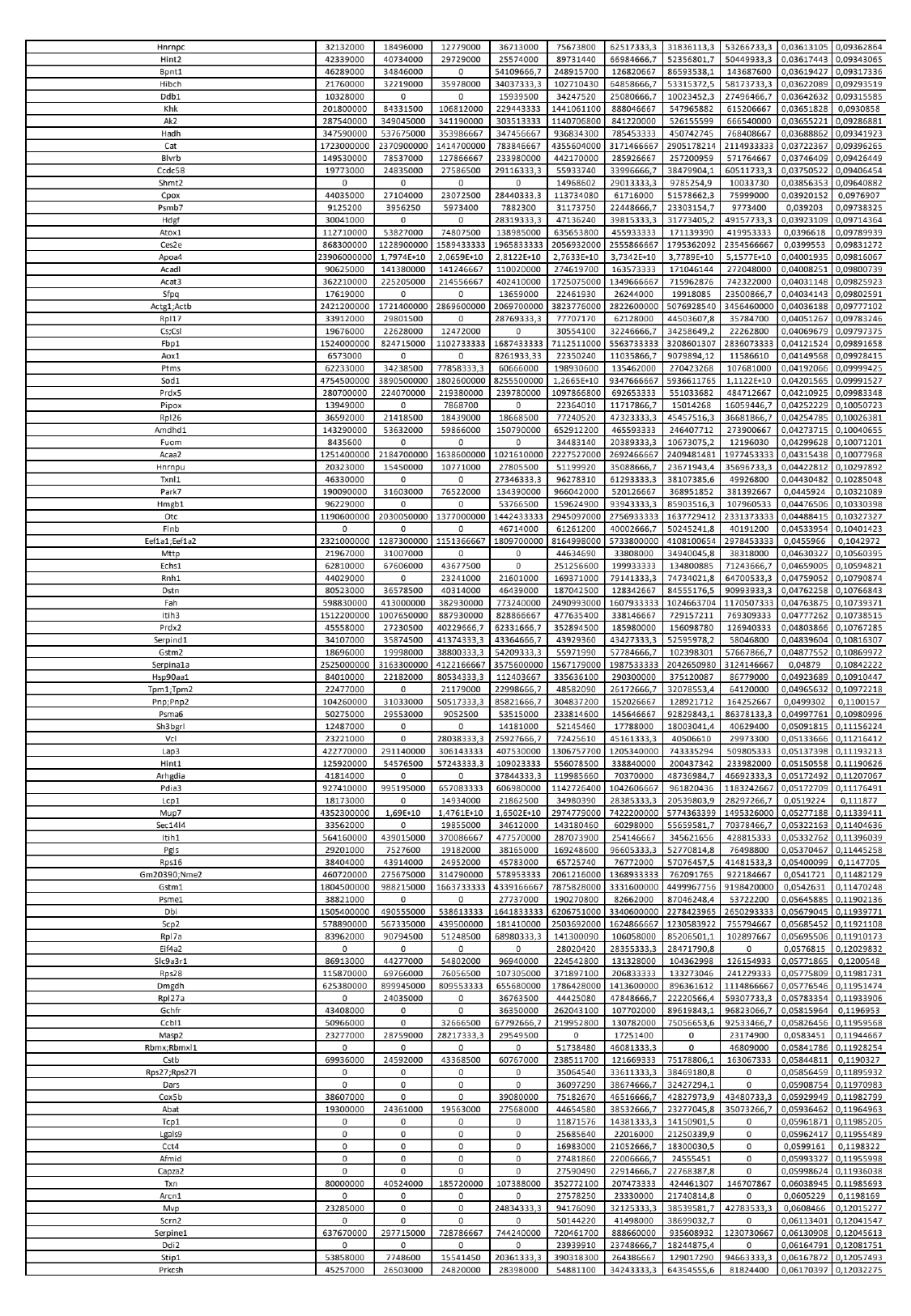

## Slide 9
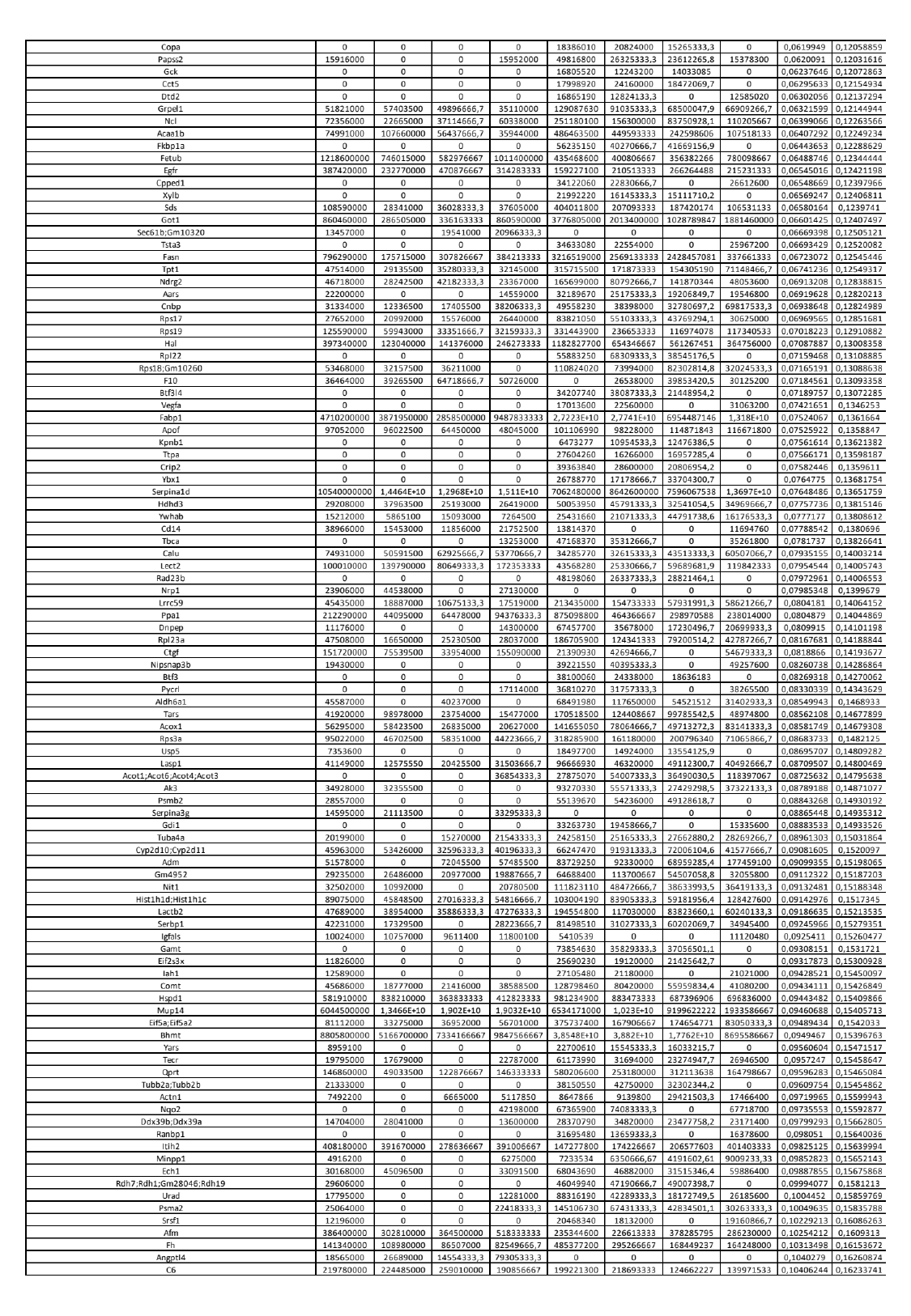

## Slide 10
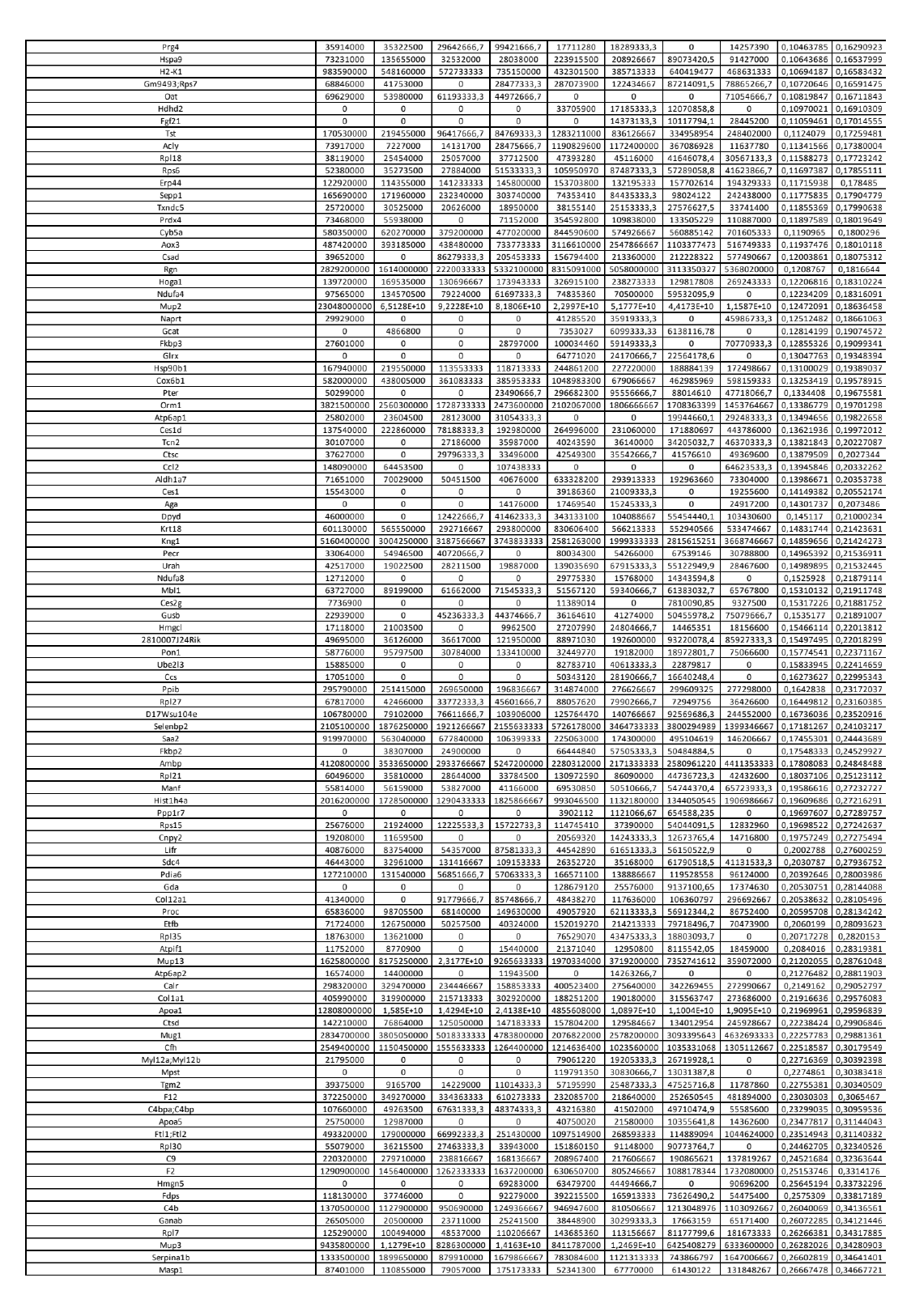

## Slide 11
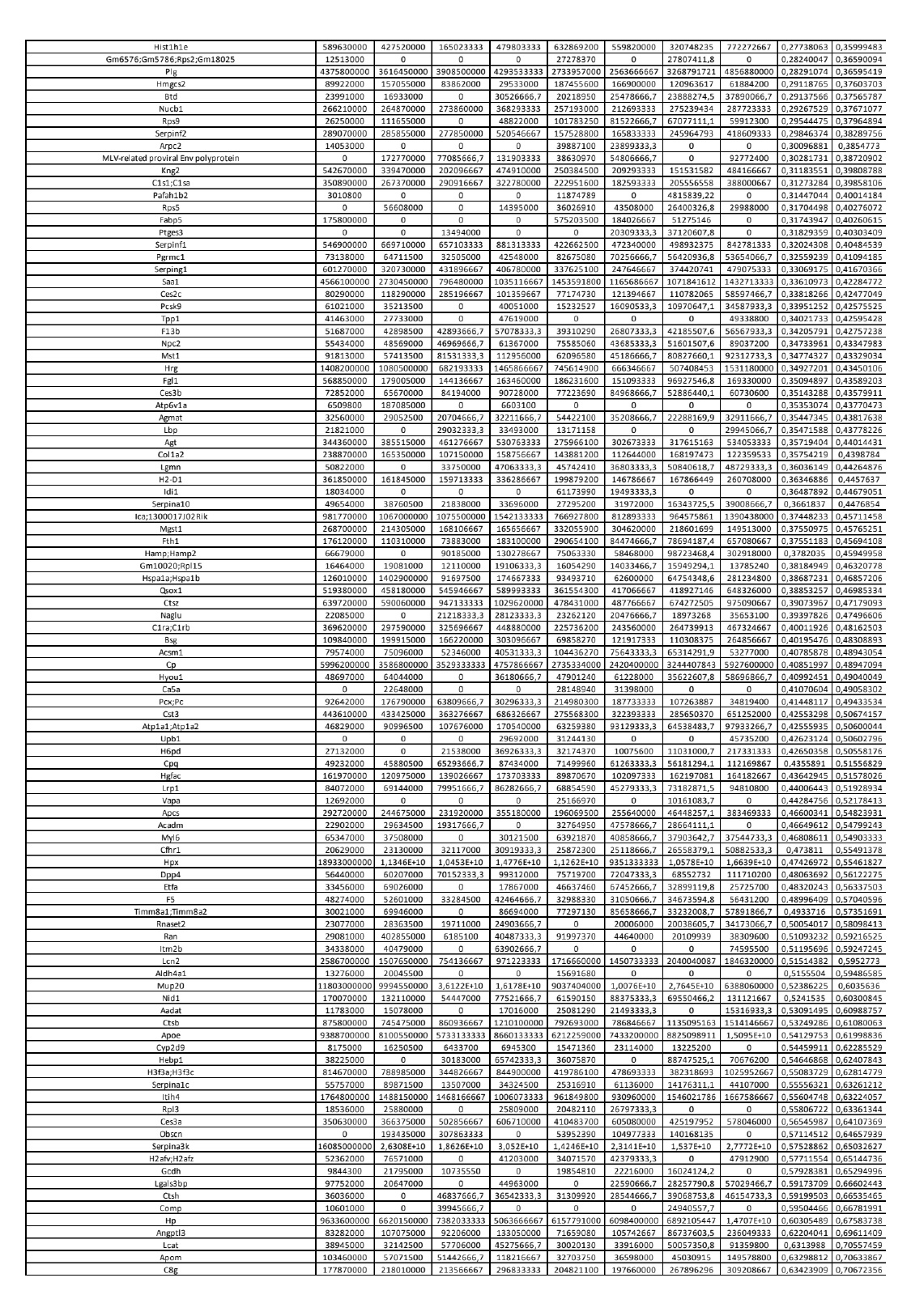

## Slide 12
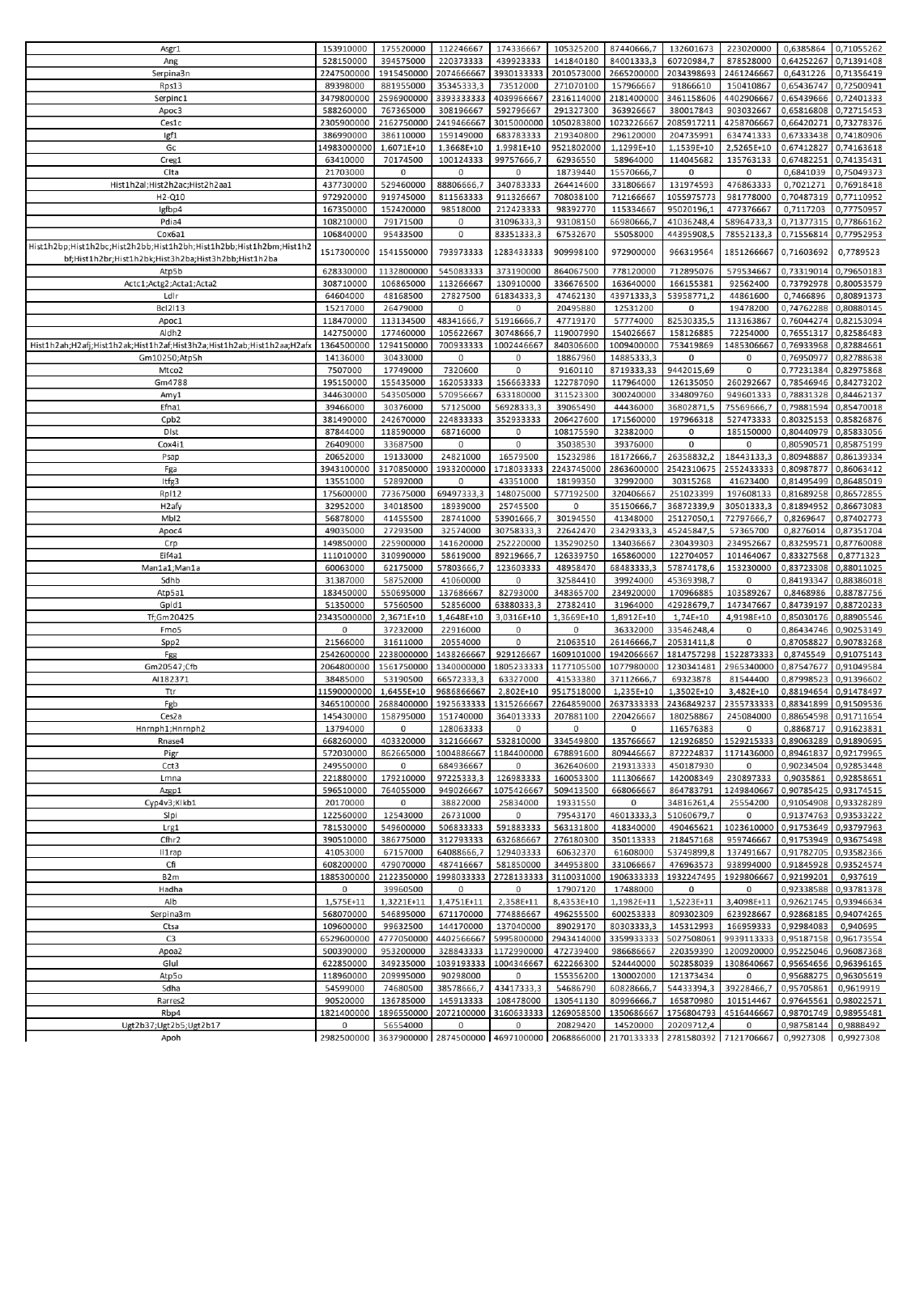

## Slide 13
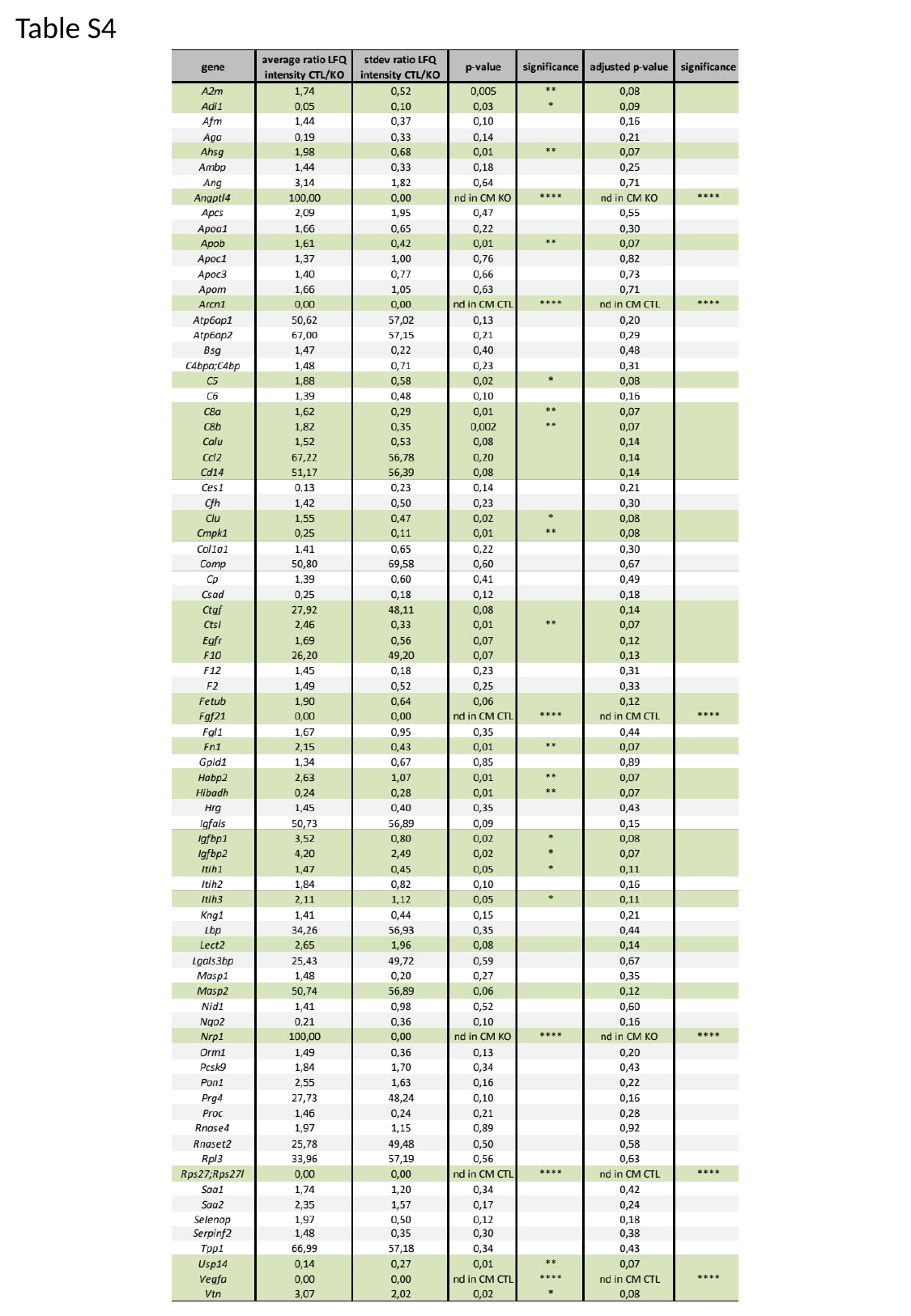

Table S4

## Slide 14
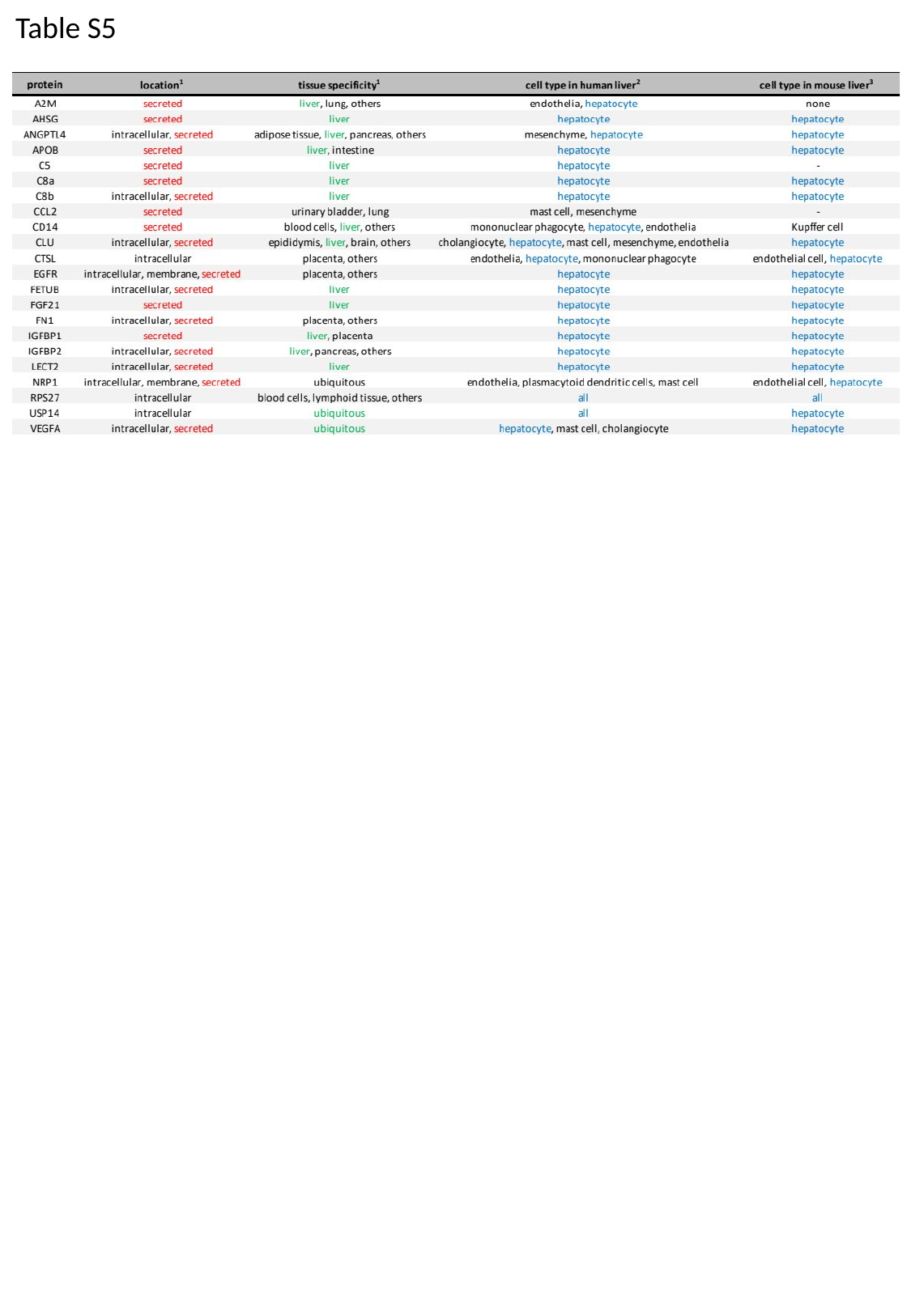

Table S5

## Slide 15
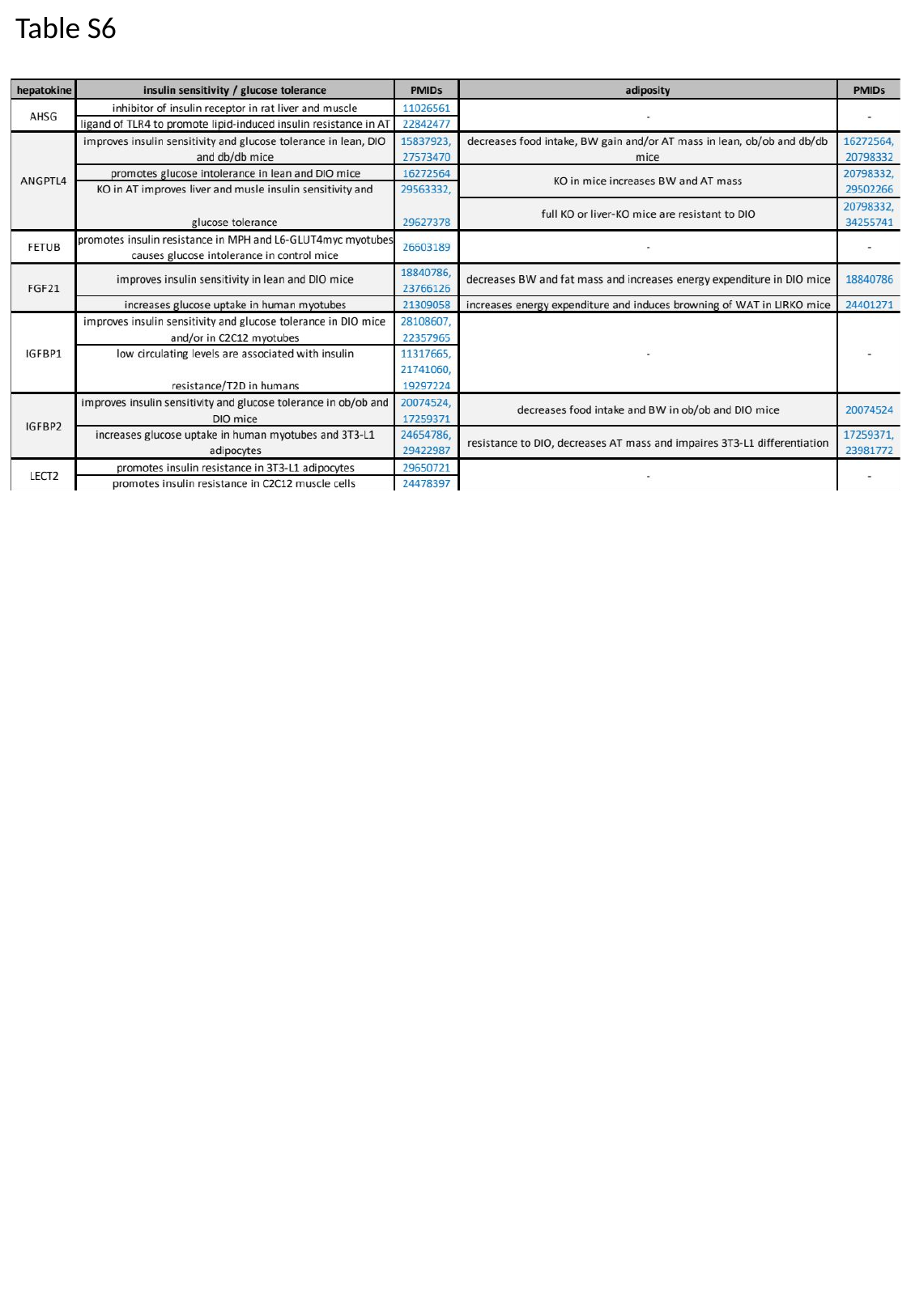

Table S6

## Slide 16
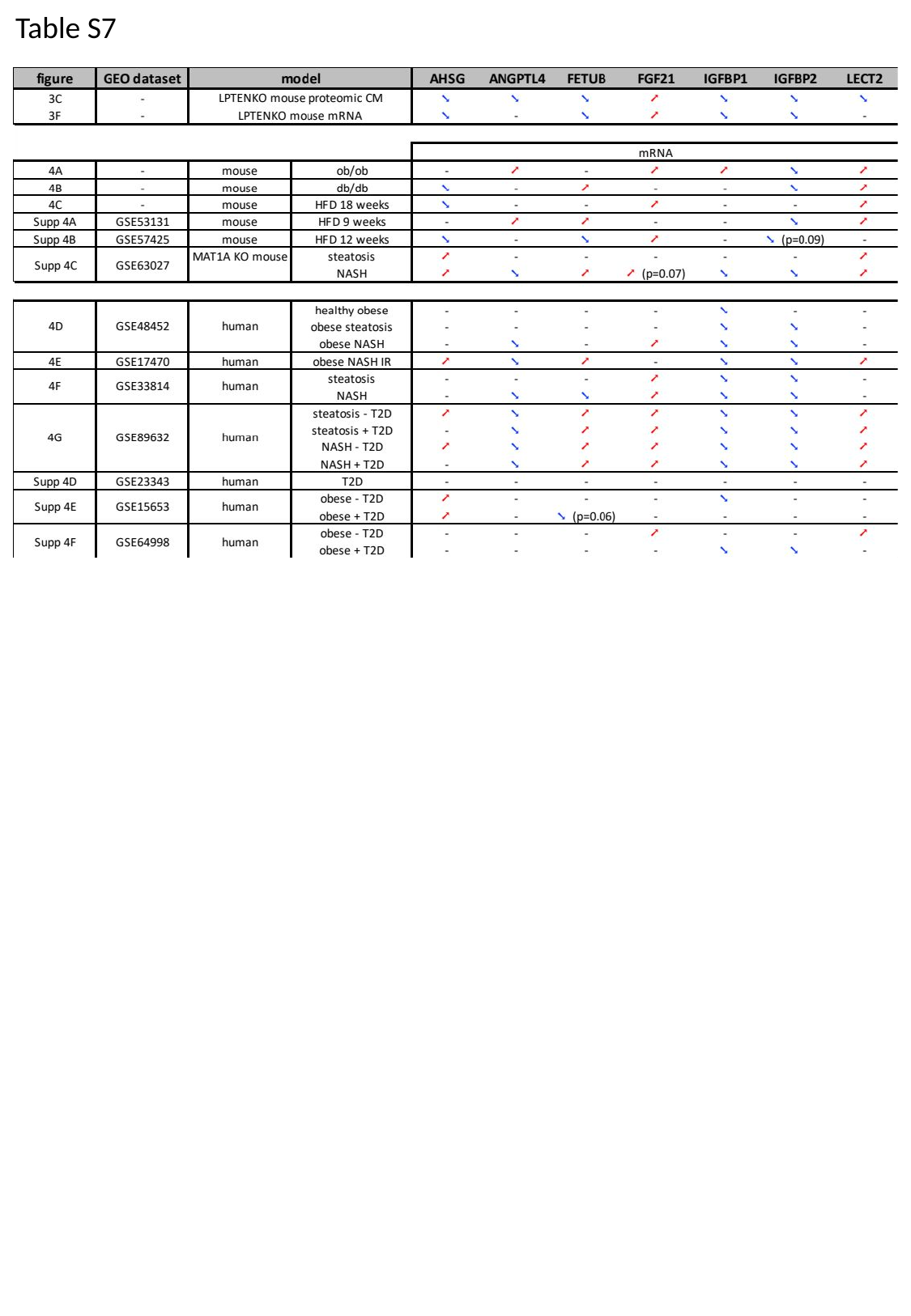

Table S7

## Slide 17
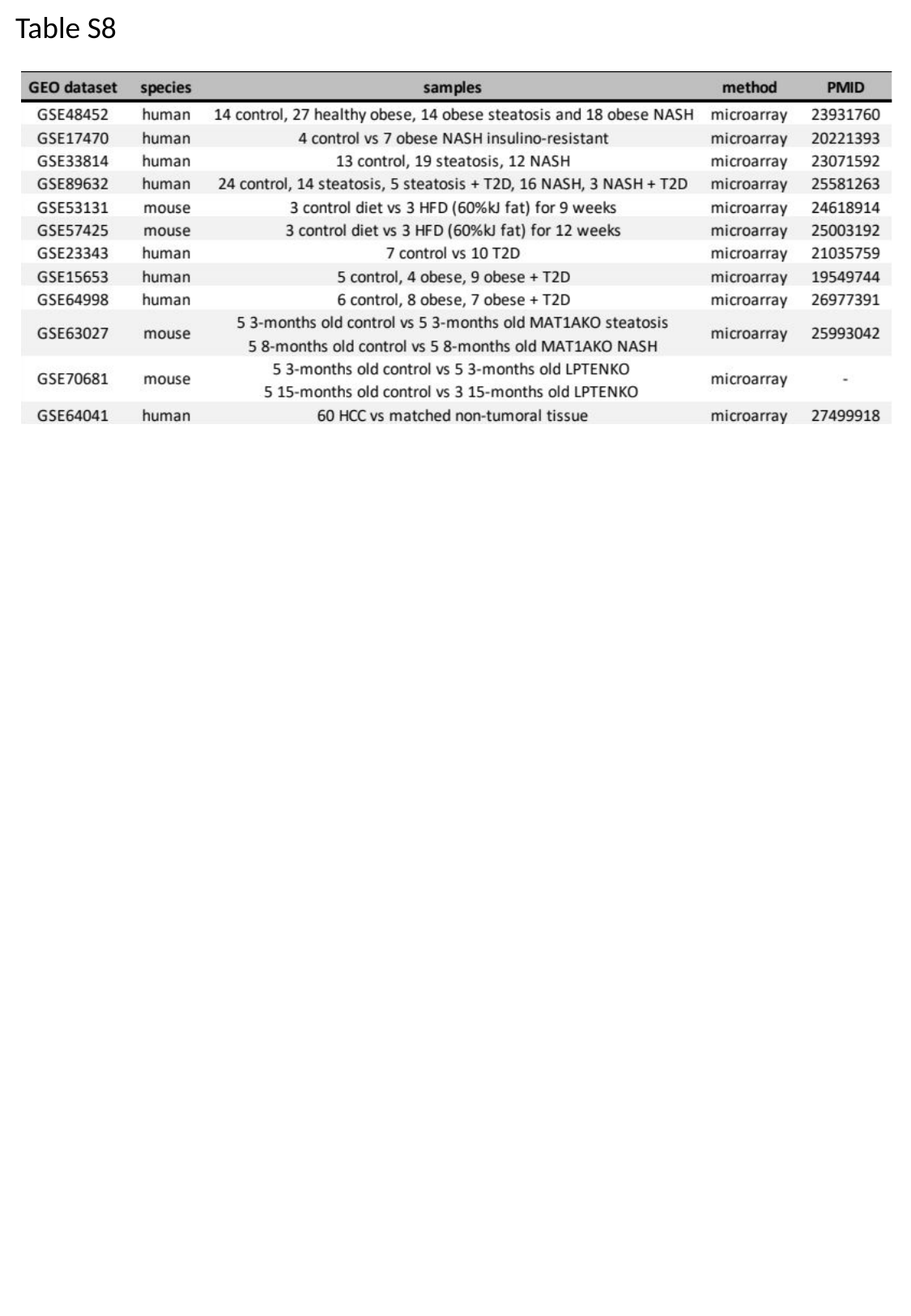

Table S8
