# Supplementary material for: Hepatic PTEN Signaling Regulates Systemic Metabolic Homeostasis through Hepatokines-Mediated Liver-to-Peripheral Organs Crosstalk
Source: Int J Mol Sci. 2022 Apr 2;23(7):3959. doi: 10.3390/ijms23073959 (PMC8999584; doi:10.3390/ijms23073959)
Supplement: Supplementary file 1 [file ijms-23-03959-s001.zip › ijms-1511495-supplementary.pdf]

Supplementary Materials and Methods

Antibodies, siRNAs, primers, kits, reagents and diets

Primary antibodies:

protein targeted	host	provider	catalogue number
ACC	rabbit	Cell Signaling Technology (MA, USA)	3662
AKT	rabbit	Cell Signaling Technology (MA, USA)	9272
β-TUBULIN	rabbit	Cell Signaling Technology (MA, USA)	2128
ERM	goat	Santa Cruz Biotechnology (TX, USA)	sc-6407
FAS	rabbit	Cell Signaling Technology (MA, USA)	3189
GLUT1	rabbit	AbD serotec	OBT1747
GLUT4	rabbit	AbD serotec	4670-1704
insulin receptor	rabbit	Santa Cruz Biotechnology (TX, USA)	sc-711
IRS1	rabbit	Santa Cruz Biotechnology (TX, USA)	sc-559
IRS2	rabbit	Cell Signaling Technology (MA, USA)	4502
p-IR Tyr1146	rabbit	Cell Signaling Technology (MA, USA)	3021
p-AKT Ser473	rabbit	Cell Signaling Technology (MA, USA)	9271
p-AKT Thr308	rabbit	Cell Signaling Technology (MA, USA)	9275
PPARG	rabbit	Santa Cruz Biotechnology (TX, USA)	sc-7196
PTEN	rabbit	Cell Signaling Technology (MA, USA)	9559
SREBP-1C	rabbit	Santa Cruz Biotechnology (TX, USA)	sc-8984
TROPONIN T	mouse	Sigma-Aldrich (MO, USA)	T6277

Secondary antibodies:

protein targeted	host	provider	catalogue number
HRP-conjugated anti-goat	rabbit	Sigma-Aldrich (MO, USA)	A5420
HRP-conjugated anti-mouse	goat	Bio-Rad (Cressier, Switzerland)	170-6516
HRP-conjugated anti-rabbit	goat	Bio-Rad (Cressier, Switzerland)	170-6515

siRNAs:

gene targeted	name	provider	catalogue number
Ahsg	Mm_Ahsg_5 FlexiTube siRNA	Qiagen (Basel, Switzerland)	SI02672264
Angptl4	Mm_Angptl4_1 FlexiTube siRNA	Qiagen (Basel, Switzerland)	SI00897883
Fetub	Mm_Fetub_1 FlexiTube siRNA	Qiagen (Basel, Switzerland)	SI01001847
Fgf21	Mm_Fgf21_2 FlexiTube siRNA	Qiagen (Basel, Switzerland)	SI01002414
Igfbp1	Mm_Igfbp1_3 FlexiTube siRNA	Qiagen (Basel, Switzerland)	SI01074101
Igfbp2	Mm_Igfbp2_1 FlexiTube siRNA	Qiagen (Basel, Switzerland)	SI00174461
Lect2	Mm_Lect2_1 FlexiTube siRNA	Qiagen (Basel, Switzerland)	SI00194712
Pten	Mm_Pten_6 FlexiTube siRNA	Qiagen (Basel, Switzerland)	SI02734494
control	AllStars Negative Control siRNA	Qiagen (Basel, Switzerland)	1027280

Mouse primers:

gene	forward	reverse
Acaca	5'-GGACACCAGTTTTGCATTGA-3'	5'-AGTTTGGGAGGACATCGAAA-3'
Acacb	5'-CACATCCTTGTGGATTACGG-3'	5'-CGATAAATCCGGTCTTCTGC-3'
Acta2	5'-AAAAAAAACCACGAGTAACAAATCAA-3'	5'-TCAGCGCCTCCAGTTCCT-3'
Adipoq	5'-TCCTGGAGAGAAGGGAGAGAAAG-3'	5'-TCATTCCAACATCTCCTGTCTCA-3'
Ahsg	5'-CCTCTCCCAGTGTCTACTC-3'	5'-AGCCATGTTGCTTCTCTG-3'
Alb	5'-TGCTTTTTCCAGGGGTGTGTT-3'	5'-TTACTTCCTGCACTAATTTGGCA-3'
Angptl4	5'-CAAAACAGCAAGATCCAGCA-3'	5'-CCTCTTTCCCCTCGAAGTCT-3'
Cebpa	5'-CAAGAACAGCAACGAGTACCG-3'	5'-GTCACTGGTCAACTCCAGCAC-3'
Cspg4	5'-AGGCGTCTACCGATGTGATGT-3'	5'-TGGCTGCCCTGTAGTGAAAC-3'
Dlk1	5'-CGAAATAGACGTTCGGGCTT-3'	5'-TCGTACTGGCCTTTCTCCAG-3'
Fabp4	5'-CACCGAGATTTCCTTCAAACTG-3'	5'-TTTCATAACACATTCCACCACC-3'
Fasn	5'-AAGTTGCCCGAGTCAGAGAACC-3'	5'-ATCCATAGAGCCCAGCCTTCCATC-3'
Fetub	5'-GGCCCTGCTTACTATGTGGA-3'	5'-TCTACGGGTTGGATCAGAGG-3'
Fgf21	5'-CAGTCCAGAAAGTCTCCTG-3'	5'-GATCAAAGTGAGGCGATCC-3'
Fgfr1	5'-TGAAGATGTTGAAGTCCGAC-3'	5'-CATAAAGAGGACCATCCTGTG-3'
Fgfr2	5'-CACCGAGAAGATGGAGAAGC-3'	5'-GTCTGACGGGACCACACTTT-3'
Fgfr3	5'-CATCCGGCAGACATACACAC-3'	5'-TTCACTTCCACGTGCTTCAG-3'
Fn1	5'-ATCTCGGAGCCATTTGTTCCT-3'	5'-CCAGGTCTACGGCAGTTGTCA-3'
Gck	5'-GCGGAGATGCTCTTTGAC-3'	5'-GTCCCACGATGTTGTTCC-3'
Gli1	5'-CAGGGTCCCAGGGTTATGG-3'	5'-AGGTCGAGGCTGGCATCAG-3'
Igfbp1	5'-CTGGACAGCTTCCACCTGAT-3'	5'-GTTGGGCTGCAGCTAATCTC-3'
Igfbp2	5'-CTTAAGCAGTGCAAGATGTCTC-3'	5'-GTTTACTGCACACTTTGGGC-3'
Lect2	5'-TAGCAGGACCATGGGCTAAC-3'	5'-GCCCACTATCTTCCCAGTGA-3'
Pdgfrb	5'-GAGGCTTATCCGATGCCTTCT-3'	5'-AGACATGTTGCGAGTAGACAAAACTAA-3'
Pkm	5'-TCGCATGCAGCACCTGATT-3'	5'-CCTCGAATAGCTGCAAGTGGTA-3'
Plin1	5'-GGTGTTACAGCGTGGAGAG-3'	5'-TGGAAGCACTCACAGGTC-3'
Plin2	5'-TAATGCCATCACCAAGTCGG-3'	5'-CTTCCATCTCCAGCTCCTC-3'
Pparg	5'-CTCACAATGCCATCAGGT-3'	5'-GCTGGTCGATATCACTGG-3'
Ppia	5'-CAAATGCTGGACCAAACACAA-3'	5'-GCCATCCAGCCATTCAGTCT-3'
Rn18s	5'-ACATCCAAGGAAGGCAGCAG-3'	5'-TTTTCGTCACTACCTCCCCG-3'
Rps9	5'-GACCAGGAGCTAAAGTTGATTGGA-3'	5'-TCTTGGCCAGGGTAAACTTGA-3'
Serpina1a	5'-CCCGGATCTTCAACAATGG-3'	5'-TTATGCACAGCCTTGCTG-3'
Slc2a1	5'-TCAATGCTGTGTTCTACTACTC-3'	5'-TCTACAACAAACAGCGACAC-3'
Slc2a4	5'-CTCATGGGCCTAGCCAATG-3'	5'-GGGCGATTTCTCCCACATAC-3'
Srebf1	5'-AAGCAAATCACTGAAGGACCTGG-3'	5'-AAAGACAAGGGGCTACTCTGGGAG-3'

Human primers:

gene	forward	reverse
AHSG	5'-TTCCAGTCCAGACTCAGC-3'	5'-CGGGAAATTTCCTCCAGC-3'
ALB	5'-CTAGAGAAGTGCTGTGCC-3'	5'-CCACGGATAGATAGTCTTCTG-3'
ANGPTLl4	5'-ACTCTAGAGGCGTGGACCAA-3'	5'-TACACACAACAGCACCAGCA-3'
FETUB	5'-CCTTTCCCCAAAGAAAAAGC-3'	5'-GCGTCCTTCACTAGCCACTC-3'
FGF21	5'-ACCTGGAGATCAGGGAGGAT-3'	5'-AGTGGAGCGATCCATACAGG-3'
IGFBP1	5'-CTGCCAAACTGCAACAAGAA-3'	5'-TATCTGGCAGTTGGGGTCTC-3'
IGFBP2	5'-CCTCAAGTCGGGTATGAAGG-3'	5'-ACCTGGTCCAGTTCCTGTTG-3'
LECT2	5'-GAGTGTGGGGGCAACTCTAA-3'	5'-TGGTGAGGCCTCTGACTTCT-3'
SERPINA1	5'-CTTCTTTAAAGGCAAATGGGAG-3'	5'-CTGGACAGCTTCTTACAGTG-3'

Kits, reagents and diets:

name	provider	catalogue number
collagenase	Sigma-Aldrich (MO, USA)	C5138
complete EDTA-free inhibitors	Roche (Basel, Switzerland)	11873580001
dexamethasone	Sigma-Aldrich (MO, USA)	D8893
ECL advance	Amersham (Dübendorf, Switzerland)	RPN2135
glucose 20%	Sintetica-Bioren (Couvet, Switzerland)	-
glucotrend active	Roche (Basel, Switzerland)	4454308
High Capacity RNA-to-cDNA kit	Thermo Fisher Scientific (MA, USA)	4387406
insulin (Humalog)	Eli Lilly (Vernier, Switzerland)	-
isofluorane	Rothacher & Partner (Berne, Switzerland)	ISO250
nitrocellulose membranes	Amersham (Dübendorf, Switzerland)	RPN303D
penicillin/streptomycin	Invitrogen (CA, USA)	15140122
percoll	GE Healthcare (Uppsala, Sweden)	17-0891-01
Pierce BCA Protein Assay kit	Thermo Fisher Scientific (MA, USA)	23225
recombinant mouse FGF21	R&D Systems (MN, USA)	8409-FG
sodium pyruvate	Sigma-Aldrich (MO, USA)	P5280-25G
SYBR Select Master Mix	Thermo Fisher Scientific (MA, USA)	4472920
triglyceride kit	Roche/Hitachi (Rotkreuz, Switzerland)	12016648-122
TRIzol Reagent	Thermo Fisher Scientific (MA, USA)	15596-018
viromer blue	BioNTech Delivery Technologies GmbH (Halle, Germany)	VB-01LB-00
William's E Medium, DMEM, RPMI, PBS and others culture media	Thermo Fisher Scientific (MA, USA)	-
chow diet (5% cal/fat)	ssniff Spezialdiäten GmbH (Soest, Germany)	E15000-04, EF R/M Control
high fat diet (60% cal/fat)		E15741-34, EF D12492 (II) mod. 60 kJ% Fat

Metabolic phenotyping of LIPTENKO mice

For glucose and pyruvate tolerance tests (GTT and PTT), mice were intraperitoneally (IP) injected with glucose at 1.5 g/kg of body weight or with pyruvate at 2 g/kg of body weight after overnight starvation. Glucose levels were measured from blood samples at different times using a glucometer. Lean and fat body masses were measured by EchoMRI (EchoMRI-700 quantitative nuclear magnetic resonance analyzer, Echo Medical Systems, TX, USA).

Histology

Liver tissues were fixed overnight in 4% paraformaldehyde and washed twice with PBS. Then, they were dehydrated, embedded in paraffin and 5 µm thick sections were stained with hematoxylin/eosin (H&E). To stain neutral lipids, livers tissues were embedded and frozen with liquid nitrogen and 2-methyl-butane in OCT. 5 µm thick cryosections were fixed with 4% paraformaldehyde for 15 minutes at room temperature and stained with Oil Red O.

Determination of hepatic triglyceride content

Hepatic triglyceride (TG) content was measured by hexane/isopropanol extraction followed by a colorimetric enzymatic assay using a commercial kit (Roche/Hitachi, Rotkreuz, Switzerland) as previously described [34]. TG content was normalized by the liver piece weight.

Isolation and treatments of mouse primary hepatocytes

Mouse primary hepatocytes (MPH) were isolated as previously described [35] and cultured in William's E Medium supplemented with 10% FBS, 1 µM dexamethasone, 1 nM insulin, 1% penicillin/streptomycin, 0.25% gentamicin and 1% L-glutamine. Treatments with inhibitors or drugs were done 5 hours after seeding for 24 hours. Upon discovery, the IC50 for the classical PI3K inhibitor LY294002 was calculated at 1.4 µM using 50 µM of LY294002 in human neutrophils [165]. For PD98059, a MEK inhibitor, the first reported IC50 was 2-7 µM for MEK1 and 50 µM for MEK2 with 50 µM of PD98059 in 3T3-L1 cells [166] and for Compound C, an AMPK inhibitor, the Ki was 109 nM and the significant effect in primary rat hepatocytes was achieved at 20 and 40 µM [167]. Incubations with insulin or glucose were realized on overnight-deprived MPH (cultured with medium without insulin and supplemented with 0.5% BSA instead of 10% FBS) for 6h.

Muscle, adipocyte and hepatic cell line culture

Undifferentiated C2C12 and 3T3-L1 cells were cultured in DMEM supplemented with 10% FBS and 1% penicillin/streptomycin. One day post seeding, 10% FBS was replaced by 2% horse serum for 5-6 days to differentiated C2C12 muscle cells. C2C12 cells were then cultured with conditioned media or with different concentrations of recombinant mouse FGF21 for 24h before insulin stimulation (10 nM, 10 minutes) or glucose uptake experiments. To initiate differentiation of 3T3-L1 to adipocyte-like cells, 10 µg/ml insulin, 0.5 mM IBMX, 0.25 µM dexamethasone and 10 µM rosiglitazone were added in conditioned media. 4 days later, only insulin was added for another 4 days, where differentiation was reached. Human (THLE-2, Huh-7, HepG2, Hep3B, HepaRG and SNU-398) and mouse (AML12 and Hepa1-6) hepatic cell lines were cultured as previously described [35].

Glucose uptake

Differentiated C2C12 cells were incubated with conditioned media for 24h, washed three times with warm Krebs-Ringer Hepes buffer (KRB) (135 mM NaCl, 5 mM KCl, 1 mM MgSO4, 0.4 mM K2HPO4, 20 mM Hepes, 0.5% BSA, pH 7.4) and then incubated with KRB at 37°C for 15 min. Insulin (100 nM) or cytochalasin B (10µM), a GLUTs inhibitor used as negative control, were added for 20 min before addition of 3H-deoxyglucose 0.2 µCi/ml in 20 µM of non-radioactive deoxyglucose for 20 min at 37°C. Cells were washed three times with ice-cold KRB buffer and lysed in NaOH 0.25N for 5 min. Liquid scintillator was added and the β particles emission was detected using an appropriate counter (Wallac 1409 Liquid Scintillation Counter). Glucose uptake was normalized by protein quantity.

Lipid staining in 3T3-L1 adipocytes

3T3-L1 cells were incubated for 30 min at room temperature in the dark with 1 µg/ml BODIPY (boron-dipyrromethene) 493/503 (Molecular Probes) and Hoechst 33342 in order to visualize lipid droplets and nuclei, respectively. Pictures were taken with EVOS FL imaging system (Advanced Microscopy Group, Life Technology, Mill Creek, WA, USA). Fluorescence and cell number quantification were done with ImageJ software.

Bioinformatics Analysis

Metabolomic and proteomic integration: Our proteomic analysis of the liver of control and LPTENKO mice has been recently published [35]. Integration of metabolomic and proteomic was performed using MetaboAnalyst 4.0 Joint Pathway Analysis module [168]. Metabolite and protein lists were constructed based on adjusted p-value < 0.15 and p-value ≤ 0.05, respectively and fold change ≤ 0.67 or ≥ 1.5 criteria. Lists, together with their log2 fold change values, were imported to the module. The analysis was performed against the "Metabolic pathways (integrated)" database using the hypergeometric test for enrichment analysis, degree centrality as topology measure and with combining p-values at the pathway level. Pathways at false discovery rate (FDR) < 0.05 were considered significantly enriched.

Identification of predicted human secreted proteins in the proteomic analysis: In order to identify circulating proteins relevant in humans among the deregulated proteins obtained from proteomic analysis, our proteomic data were crossed with a list of

predicted human secreted proteins, *i.e.* proteins with a signal peptide, established from three different prediction methods: SignalP 4.0 (http://www.cbs.dtu.dk/services/SignalP-4.0/), Phobius (http://phobius.sbc.su.se/) and SPOCTOPUS (http://octopus.cbr.su.se/).

Identification of proteins involved in metabolism: To identify proteins potentially involved in lipid and/or glucose metabolism and/or related diseases in human, cross analysis were done between our potential secreted candidates and lists of genes associated with lipid metabolism (776), glucose metabolism (3402), obesity (1899) and type 2 diabetes / insulin resistance (3516), established using MetaCore database (version 6.33 build 69110) (https://portal.genego.com/).

Gene Expression Omnibus (GEO) analysis: The relative mRNA expression of hepatokines of interest in the liver of obese, type 2 diabetes and/or NAFLD patients or mice was assessed thanks to the public available database "Gene Expression Omnibus (GEO)" (https://www.ncbi.nlm.nih.gov/gds). Only series including a control group and analyzed with the GEO2R algorithm were considered. In case where different probes were found for one gene, only the most significant one was considered.

Survival analyses: Overall survival of HCC patients were obtained from the GEPIA database (http://gepia.cancer-pku.cn/). Survival analyses were represented with Kaplan-Meier survival curves. For each gene of interest, patients were segregated in low and high expressing groups with the following cutoffs: 80% for the high expressing group and 20% for the low expressing group.

Supplementary Figure legends

Figure S1: *In vitro* treatment of C2C12 muscle cells and 3T3-L1 adipocytes with conditioned media from primary hepatocytes.

(A) Bright-field pictures of mouse primary hepatocytes (MPH) from 4-months old Pten$^{lox/lox}$ (CTL) and LPTENKO mice. Conditioned media (CM) were collected 24h after MPH adhesion and incubated on differentiated C2C12 muscle cells for 24h before insulin stimulation for western blot analyses and glucose uptake measurements. **(B)**

Representative western blot analyses of insulin receptor substrate (IRS) 1 and 2, glucose transporter (GLUT) type 1 and 4 and TROPONIN T protein expression in C2C12 cells incubated with CM CTL and KO and quantifications. Values are means ± SEM of 4-6 independent experiments. **(C)** Representative western blot analyses of glucose transporter (GLUT) type 1 and 4 and β-TUBULIN protein expression in soleus muscle of 4-months old control (CTL) and LPTENKO mice and quantifications. Values are means ± SEM of 6 mice per group. **(D)** Bright-field pictures of 3T3-L1 adipocytes, cultured in normal media, before and after 8 days of differentiation.

Figure S2: Metabolic features of liver-specific inducible PTEN KO mice.

Body weight, liver weight and liver/body weight ratio **(A)**, body composition by echoMRI **(B)**, glucose tolerance test (GTT) and area under the curve (AUC) **(C)**, pyruvate tolerance test (PTT) and area under the curve (AUC) **(D)**, representative liver histology (Oil Red O staining) **(E)** and hepatic triglyceride content **(F)** of 5-months old $Pten^{lox/lox}$ (CTL) and AlbCre-$ERT2^{Tg/+}/Pten^{lox/lox}$ (LIPTENKO) mice treated with tamoxifen 3 months prior to analyses. **(G)** Relative mRNA expression of hepatokines by RT-qPCR in the liver of control (CTL) and LIPTENKO mice. *Ppia* was used as reference gene to normalize the RT-qPCR analyses. Values are means ± SEM of 4-5 mice per group. *: $p \leq 0.05$, **: $p \leq 0.01$, ***: $p \leq 0.001$, ****: $p \leq 0.0001$ (two-way ANOVA for glycemia 2C, D and t-test for the others).

Figure S3: Metabolic features of different genetically obese mouse models.

Representative liver histology (H&E staining) **(A)** and body weight **(B)** of ob/ob and db/db mice and their respective controls. Values are means ± SEM of 5-6 mice per group. ****: $p \leq 0.0001$ (t-test).

Figure S4: Hepatokines expression in the liver of obese mouse models with IR and steatosis and in obese, type 2 diabetes and/or NAFLD patients.

Relative mRNA expression of hepatokines in the liver of: mice fed with high-fat-diet (HFD) for 9 **(A)** and 12 **(B)** weeks, methionine adenosyltransferase 1A (MAT1A) knockout mice **(C)** and several cohorts of obese and/or type 2 diabetes (T2D) patients **(D**, **E** and **F)** obtained from public Gene Expression Omnibus (GEO) datasets. See Table S8 for details. *: $p \leq 0.05$, **: $p \leq 0.01$, ***: $p \leq 0.001$, ****: $p \leq 0.0001$ (t-test).

Figure S5: Hepatokines expression in isolated mouse primary hepatocytes (MPH) differentiating after seeding.

Relative mRNA expression of hepatic differentiation markers **(A)** and hepatokines **(B)** by RT-qPCR in control (CTL) and LPTENKO hepatic tissues and isolated primary hepatocytes prior (pellet) or after plating from day 1 to day 5. *Rn18S* was used as reference gene to normalize the RT-qPCR analyses. * indicates statistical difference between CTL and LPTENKO. Values are means ± SEM of 3 independent experiments. *: $p \leq 0.05$, **: $p \leq 0.01$ (t-test).

Figure S6: Validation of siRNAs efficiency in mouse primary hepatocytes.

Relative mRNA expression of hepatokines by RT-qPCR in LPTENKO (for *Fgf21*) or control (for other hepatokines) MPH 48h after transfection with siRNA scramble (siCTL) or specific siRNAs targeting each hepatokine. *Ppia* was used as reference gene to normalize the RT-qPCR analyses. Values are means ± SEM of 3 independent experiments. *: $p \leq 0.05$, **: $p \leq 0.01$, ***: $p \leq 0.001$, ****: $p \leq 0.0001$ (t-test).

Figure S7: Hepatokines expression in isolated mouse primary hepatocytes versus mouse cultured hepatic cell lines.

Relative mRNA expression of hepatokines **(A)** and hepatic differentiation markers **(B)** by RT-qPCR in mouse primary hepatocytes (MPH), AML12 and Hepa1-6 cells. *Rn18s* was used as reference gene to normalize the RT-qPCR analyses. Values are means ± SEM of 3-4 independent experiments. **: $p \leq 0.01$, ***: $p \leq 0.001$, ****: $p \leq 0.0001$ (one-way ANOVA).

Figure S8: Hepatokines expression in isolated human primary hepatocytes versus human cultured hepatic cell lines.

Relative mRNA expression of hepatokines **(A)** and hepatic differentiation markers **(B)** by RT-qPCR in human primary hepatocytes (HPH), THLE-2, Huh-7, HepG2, Hep3B, HepaRG and SNU-398 cells. Values are means ± SEM of 3-4 independent experiments. *: $p \leq 0.05$, **: $p \leq 0.01$ (one-way ANOVA).

Figure S9: Hepatokine expression in isolated mouse primary hepatocytes depleted of PTEN by siRNAs.

Representative western blot analyses of phosphatase and tensin homolog (PTEN), phosphorylated AKT (serine) and ezrin/radixin/moesin (ERM) protein expression and relative mRNA expression of hepatokines by RT-qPCR in control mouse primary hepatocytes (MPH CTL) transfected with siRNA scramble or siRNA targeting specifically *Pten* (siPten). *Ppia* was used as reference gene to normalize the RT-qPCR analyses. Values are means ± SEM of 3 independent experiments. For each gene, the mean of control group (MPH CTL transfected with siRNA scramble) = 1 and it is not represented on the graph. *: $p \leq 0.05$, **: $p \leq 0.01$ (t-test).

Figure S10: Gene expression of FGF receptors in the soleus muscle of LPTENKO mice.

Relative mRNA expression of the fibroblast growth factor receptors (*Fgfr*) 1-3 by RT-qPCR in soleus muscle of 4-months old control (CTL) and LPTENKO mice. *Ppia* was used as reference gene to normalize the RT-qPCR analyses. Values are means ± SEM of 4-5 animals. *: $p \leq 0.05$, **: $p \leq 0.01$, ***: $p \leq 0.001$ (t-test).

Figure S11: Proliferation markers expression in the liver of 3- and 15-months old LPTENKO mice.

Relative mRNA expression of proliferation markers in the liver of 3- and 15-months old LPTENKO mice obtained from public Gene Expression Omnibus (GEO) datasets. See Table S8 for details. *: $p \leq 0.05$, ***: $p \leq 0.001$ (t-test).

Figure S12: Hepatokines expression in human HCC and associated survival curves.

(A) Relative mRNA expression of hepatokines in human HCC tissues obtained from public Gene Expression Omnibus (GEO) datasets. See Table S8 for details. The noteworthy percent of patients exhibiting decreased (fold change ≤ 0.66, blue) or increased (fold change ≥ 1.5, red) hepatokines level is indicated. **(B)** Patients' survival curves as a function of low versus high hepatokines expression in tumour (data obtained from the GEPIA database in march 2020). *: $p \leq 0.05$, ***: $p \leq 0.001$ (logrank test (Mantel-Cox test)).

Supplementary Table legends

Table S1: Metabolomic analysis of the plasma from control and LPTENKO mice. Circulating amino acids, biogenic amines, acylcarnitines, lysophosphatidylcholines, phosphatidylcholines, sphingomyelins, hexoses and biles acids were measured in the plasma of 4-months old control (CTL) and LPTENKO mice (n=4). *: $p \leq 0.05$, **: $p \leq 0.01$, ***: $p \leq 0.001$ (t-test). The 13 candidates with both fold change (≤ 0.67 or ≥ 1.5) and an adjusted p-value < 0.15 are highlighted in green.

Table S2: Contribution of deregulated metabolites and proteins from LPTENKO mice on metabolic pathways.
19 significantly enriched processes are shown following the integration analysis performed between the metabolomic of plasma from CTL vs LPTENKO and the proteomic of liver from CTL vs LPTENKO mice using MetaboAnalyst 4.0. “Hits cmpd (compound)” is for metabolites.

Table S3: Proteomic analysis of conditioned media from control and LPTENKO primary hepatocytes.
A proteomic analysis of conditioned media from control and LPTENKO primary hepatocytes was performed by LC-MS/MS analysis (n=4). Label-free quantification (LFQ) values are indicated for each of the 780 detected protein. p-values were calculated by a t-test and adjusted p-value by the false discovery rate (FDR, Benjamini and Hochsberg method).

Table S4: Differentially expressed proteins with classical signal peptide for secretion in conditioned media from LPTENKO hepatocytes.
291 deregulated proteins were identified in the conditioned media (CM) of LPTENKO hepatocytes compared to CM from control (CTL) hepatocytes following liquid chromatography - mass spectrometry analysis. Among them, 80 have a signal peptide and their expressions are indicated as a ratio of untargeted label-free quantification (LFQ) intensity of CTL / KO. *: $p \leq 0.05$, **: $p \leq 0.01$, ****: $p \leq 0.0001$ (t-test). The 35 candidates with an adjusted p-value < 0.15 are highlighted in green.

Table S5: Location and tissue specificity of differentially expressed proteomic factors involved in obesity/IR/T2D and the glucose/lipid metabolism.

[1] The human protein atlas: https://www.proteinatlas.org

[2] The human liver single cell atlas: http://www.livercellatlas.mvm.ed.ac.uk

[3] The mouse liver single cell atlas: https://tabula-muris.ds.czbiohub.org

These three databases were accessed on 03 april 2020.

Table S6: Literature reviewing of the role of AHSG, ANGPTL4, FETUB, FGF21, IGFBP1, IGFBP2 and LECT2 on insulin sensitivity, glucose tolerance and adiposity.

The table reports the PMIDs of the papers used to build the sketch of Figure 3E.

Table S7: Summary of hepatokines mRNA alterations in human and mouse transcriptomic datasets.

The table summarizes the data from Figure 4 and Figure S4.

Table S8: Transcriptomic datasets from human and/or mouse with obesity, T2D, NAFLD and HCC.

All the human and/or mouse GEO datasets used for the Figure 4D-G, Figure S4, Figure S11 and Figure S12A are listed with the samples' details, the used method and the related PMID.
